# When Shear and Interfaces Matter: In Vivo Water-in-Silicone Oil Droplet Formation during Long-Term Vitreous Tamponade

**DOI:** 10.1021/acsomega.6c02409

**Published:** 2026-06-01

**Authors:** Miroslav Veith, Monika Reháčková, Patrik Rajs, Jan Motlík, Barbora Kamenická, Petr Klusoň

**Affiliations:** † Third Faculty of Medicine, 60571 Charles University, Ruská 87, 100 00 Prague, Czech Republic; ‡ Department of Ophthalmology, University Hospital Kralovské Vinohrady and Third Faculty of Medicine, Charles University, Šrobárova 50, 100 34 Prague, Czech Republic; § Institute of Animal Physiology and Genetics, Academy of Sciences of the Czech Republic, Rumburská 89, 277 21 Liběchov, Czech Republic; ∥ 48311 Institute of Chemical Process Fundamentals of the Czech Academy of Sciences, Rozvojová 2/135, 165 00 Prague, Czech Republic; ⊥ Institute for Environmental Studies, Faculty of Science, Charles University, Benátská 2, 110 00 Prague, Czech Republic

## Abstract

Silicone oil (SO) is widely used as a long-term intraocular tamponade, yet its multiphase behavior in vivo remains incompletely understood. While oil-in-water (O/W) emulsification has been extensively studied, the potential formation of water-in-oil (W/O) droplets within the oil phase has received little attention. Here, we provide the first systematic in vivo evidence for W/O droplet formation during long-term vitreous tamponade using a controlled porcine model complemented by analysis of explanted human SO samples. W/O droplets were detected directly within the vitreous cavity, demonstrating that intraocular SO functions as a dynamic multiphase soft material rather than a strictly single continuous phase. A pronounced dependence of droplet size distributions on sampling conditions was observed. Aspiration through narrow-gauge needles induced shear-driven droplet fragmentation, whereas coaspiration of aqueous fluid during infusion generated large artifactual droplets, highlighting that explantation procedures can strongly bias ex vivo observations. Oil viscosity modulated the sensitivity of the system to shear but did not determine droplet occurrence. Complementary in vitro experiments confirmed that needle passage fragments pre-existing droplets rather than inducing de novo droplet formation. By integrating in vivo observations with in vitro phase behavior and interfacial viscoelasticity data, we establish a mechanistic framework in which shear-driven emulsification and protein-mediated interfacial stabilization govern W/O droplet formation and persistence in oil-dominated biological environments. These findings demonstrate that intraocular SO should be regarded as a confined, protein-active multiphase material system and that clinical sampling itself represents a microcapillary shear process influencing observed microstructures. Beyond ophthalmology, this work highlights the critical role of interfacial phenomena and sampling-induced artifacts in the characterization of oil-based biomaterials and soft multiphase systems in complex biological settings.

## Graphical abstract

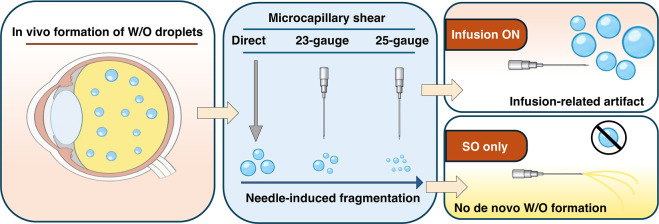

## 1 Introduction

Multiphase systems, including emulsions and dispersed droplet microstructures, are ubiquitous in soft matter and materials science and are widely exploited in applications ranging from food and cosmetics to drug delivery and advanced biomaterials.^1,2^ In many technological and biomedical contexts, controlled multiphase structuring is beneficial, enabling compartmentalization, controlled transport, and tailored mechanical or optical properties.^3,4^

In ophthalmology, however, multiphase behavior of intraocular SO is regarded as an undesirable phenomenon. SO is widely used as a long-term intraocular tamponade in vitreoretinal surgery for complex retinal detachments and proliferative vitreoretinopathy.^5,6^ Despite its established clinical benefits, SO is associated with a number of postoperative complications, including keratopathy, inflammation, elevated intraocular pressure, and secondary glaucoma.^7,8^ Many of these adverse effects have been linked to the phenomenon of SO emulsification,^9^ which has therefore been extensively studied in both clinical and experimental settings.^10–13^

From a materials perspective, intraocular SO represents a soft, oil-dominated multiphase system operating under nonequilibrium mechanical conditions in prolonged contact with a biological environment.^14,15^ Emulsification of SO is traditionally described as the formation of O/W droplets within the aqueous ocular environment.^16^ Previous studies have identified several factors contributing to this process, including mechanical shear induced by eye movements, oil viscosity, duration of tamponade, and the presence of biological surfactants such as proteins and lipids.^10,15,17,18^ However, the behavior of SO as a dynamic biphasic system within the vitreous cavity is likely more complex than currently appreciated.^18,19^

In contrast to oil-in-water emulsification,^15,20^ the potential formation of W/O droplets has received little attention in the ophthalmic literature. To date, experimental investigations of W/O emulsification have been largely restricted to in vitro model systems, including our recent work^18^ and the study of Lu et al.^21^ Our previous in vitro study^18^ demonstrated that SO-aqueous systems can form stable W/O and O/W emulsions governed by protein-mediated interfacial viscoelasticity and shear-driven emulsification,^22^ and we further hypothesized that during long-term vitreous tamponade, small amounts of aqueous fluid may enter the SO phase and become compartmentalized as W/O microdroplets stabilized by interfacial effects^23^ (Table S1, Supporting Information).

Such W/O microdroplets may incorporate dissolved proteins, ions, or inflammatory mediators and thereby modify the physical, optical, or biological properties of intraocular SO.^16^ Although several studies have examined whether prolonged intraocular residence alters the bulk physicochemical properties of SO, including analyses of explanted oil samples (e.g., Hammer et al.^24^), none of these investigations specifically addressed the presence of W/O droplets or their potential biological consequences.

Earlier clinical and experimental studies focusing on SO behavior in the eye^25–29^ do not provide direct analytical evidence for W/O droplets in explanted SO. However, the optical observations, clinical correlations, and preserved bulk physicochemical properties reported in these studies are consistent with the presence of submicron W/O microdroplets and difficult to explain by alternative mechanisms. In support of this interpretation, recent analysis of explanted intraocular SO has demonstrated a significant increase in forward light scattering in a subset of samples,^30^ a finding compatible with the presence of submicron W/O microstructures formed during early stages of emulsification.^13^

The interpretation of explanted SO samples from patients is further complicated by the possibility that droplet formation may occur during oil removal, particularly when aspiration through narrow-gauge instruments is required. Distinguishing between droplets formed during tamponade and those potentially generated during explantation is therefore essential for understanding their origin and relevance.

The present study was designed to address these knowledge gaps using a controlled porcine model of long-term vitreous SO tamponade combined with the analysis of explanted human SO samples. The primary aim was to determine whether W/O droplets form in vivo during prolonged tamponade. Secondary aims were to assess the influence of oil viscosity and aspiration technique on droplet size distribution and to compare experimental findings with observations in human samples.

## 2 Methods

### 2.1 Animals and Surgical Model

The study was conducted using two healthy juvenile domestic pigs (*Sus scrofa* domestica; Libechov minipigs;^31^ identification numbers B920 and B931; 6 months old; no known ocular pathology prior to inclusion). Animals were housed and handled in accordance with institutional and national guidelines for animal research, and all procedures were approved by the relevant ethics committee. All surgical procedures were performed under general anesthesia using standard veterinary protocols.

A standard central pars plana vitrectomy (PPV) was performed in both eyes. In contrast to routine PPV in humans, a larger amount of residual vitreous is expected to remain in the porcine eye. Following vitrectomy, a fluid–air exchange was performed as part of the standard surgical workflow. Subsequently, SO (RS-OIL ECS 5000 cS, Alchimia) was injected to achieve complete vitreous cavity tamponade. In both pigs, the right eye received SO with a viscosity of 1300 mPa·s, while the left eye received SO with a viscosity of 5000 mPa·s. After surgery, SO tamponade was maintained in all eyes for a period of two months. During this time, animals were monitored according to standard postoperative veterinary protocols.

At the end of the tamponade period, SO samples were collected from each eye prior to eye removal. To minimize artifactual emulsification during sampling, all SO samples were intentionally collected with the intraocular infusion turned off. Sampling was performed sequentially from each eye as follows: (i) aspiration through a 23-gauge needle; (ii) aspiration through a 25-gauge needle; (iii) the third and final sample consisted of SO obtained by direct extraction without the use of a needle or capillary after animal euthanasia, enucleation of both eyes and subsequent incision of the globes. Care was taken to avoid additional mechanical agitation during sample handling.

A bilateral design with different SO viscosities minimized interanimal variability. Central PPV without retinal ablation reduced inflammation-related confounders, and SO sampling without active infusion minimized artifactual W/O droplet formation. All sclerotomies were self-sealing and unsutured. No adverse ocular events or complications were recorded during the tamponade period. The surgical procedures were completed without intraoperative complications in all eyes.

### 2.2 Human Silicone Oil Samples

Totally eight SO samples were obtained from patients undergoing PPV. All human SO samples were obtained from female patients aged 28–84 years. Clinical history and perioperative data were collected but are not analyzed in the present study. All procedures were performed as part of routine clinical care. In all cases, SO (RS-OIL ECS, Alchimia) with a viscosity of 5000 mPa·s was used as a long-term intraocular tamponade prior to explantation.

SO was removed from the vitreous cavity using a Constellation Vision System (Alcon, Fort Worth, TX, USA). A dedicated vacuum syringe equipped with a blunt capillary needle was inserted into the vitreous cavity through a pars plana port, and SO was aspirated under controlled conditions.

For each patient, three separate SO samples were collected sequentially using different aspiration conditions (i) aspiration through a 25-gauge needle (approx. 0.5 mL of sample): with the infusion turned off (ii) aspiration through a 23-gauge needle (approx. 0.5 mL of sample); with the infusion turned off; and (iii) aspiration with the intraocular infusion turned on, following standard clinical explantation practice. Following aspiration, each syringe containing the SO sample was sealed with a plastic cap and stored at ambient conditions until further analysis.

### 2.3 In Vitro Experiments

#### 2.3.1 Aspiration Experiments

Model W/O emulsions containing large droplets were prepared under controlled laboratory conditions. Emulsification parameters were selected according to our previous in vitro study.^18^ SO (medical-grade, viscosity 1300 mPa·s; Bausch and Lomb, USA) and aqueous phase (model aqueous humor; the composition is detailed in ref 18) were placed into glass vial (inner dimensions: diameter 25 mm, height 40 mm; 20 mL in volume) in weight ratio 9:1 to a total weight of 10 g. The mixtures were shaken within 24 h. at ambient temperature in circular motion at 200 rpm using the laboratory shaker GLF 3005 equipped with a vial holder.

The emulsions were aspirated using syringe pump at a constant flow rate through either a 23-gauge (diameter 0.6 mm) or a 25-gauge (diameter 0.5 mm) needle using syringe (total volume 10 mL). Aspiration at a constant flow rate was chosen to allow controlled comparison between needle gauges. Each sample was aspirated once to avoid cumulative shear effects. Immediately after aspiration, the emulsions were analyzed by optical microscopy. Droplet morphology and size distributions before and after aspiration were evaluated to assess shear-induced droplet breakup. To assess whether sampling alone could induce W/O droplet formation, control experiment was performed. Pure SO without added aqueous phase was aspirated under identical flow conditions and analyzed microscopically to verify the absence of droplet formation.

#### 2.3.2 Infusion-Mimicking Experiment

To assess the potential effect of infusion-related flow conditions on droplet formation, a simplified in vitro model experiment was performed. SO (5000 mPa·s, 4 mL) was placed in a round-bottom vessel equipped with a side neck. An aqueous KCl solution was introduced into the oil phase through a capillary using a syringe pump at a constant flow rate. During continuous aqueous inflow, samples of the oil phase were simultaneously withdrawn and analyzed by optical microscopy. This setup was designed to qualitatively mimic the copresence of incoming aqueous fluid and oil sampling under infusion-like conditions. The experiment was performed as a proof-of-principle model to evaluate whether such conditions can promote the formation of W/O droplets.

### 2.4 Microscopy, Image and Data Analysis

All SO samples were analyzed at 100× magnificence using an optical microscope (ME-2665; Euromex GmbH, Duiven, The Netherlands) equipped with an objective (S.PlanM 10×/0.25) and a digital camera (Nikon D300s) mounted via a G-mount adapter. Images were acquired using DigiCam Control software. Due to the spatial resolution of the images (3.6 pixels/μm), droplets smaller than 1.1 μm were excluded from size distribution analysis. Methylene orange was used as a water-soluble dye to selectively stain the aqueous phase and confirm the presence of W/O droplets; the description of this identification method is detailed in ref 18.

Droplet size, count, and size distributions were determined using ImageJ software (version 1.54g, NIH, USA) using a standardized image analysis workflow^32^ across all data sets to ensure comparability of results (see Supporting Information for details). For each sample, multiple microscopy images were acquired (six images per sample; Figure S1 in Supporting Information) to capture representative regions of the droplet population. These images were combined to construct droplet size distributions for each individual sample.

In addition to histogram-based size distributions, quantitative measures of intrasample variability were determined from the same image sets. Specifically, all optically detectable droplets identified across the six images were pooled for each sample, and mean droplet diameter ± standard deviation was calculated from the resulting droplet-level measurements. To further assess spatial heterogeneity within individual samples, the number of droplets and mean droplet diameter were also determined separately for each image and expressed as mean ± standard deviation across the six microscopy fields.

It should be noted that these measurements represent technical replicates within a single sampling event rather than independent biological replicates. Therefore, the presented size distributions are intended to provide a representative characterization of droplet populations rather than a statistical analysis of intersample variability. Droplet size distributions were evaluated from all acquired images and are presented as histograms showing droplet intensity (%) as a function of droplet diameter. Droplet intensity was calculated as the relative fraction of droplets within a given diameter range.

## 3 Results and Discussions

### 3.1 Porcine Model

W/O droplets were detected in all four eyes after long-term SO tamponade, although not necessarily in every individual sample obtained from a given eye. Representative microscopy images from animal no. 931 (SO 5000 mPa·s) are shown in Figure 1A–C. The aqueous nature of the droplets was independently confirmed by methylene orange staining (Figure 1D). Complete droplet size distributions for both animals and both viscosities are provided in Figures S2 and S3 in Supporting Information.

**Figure 1:**
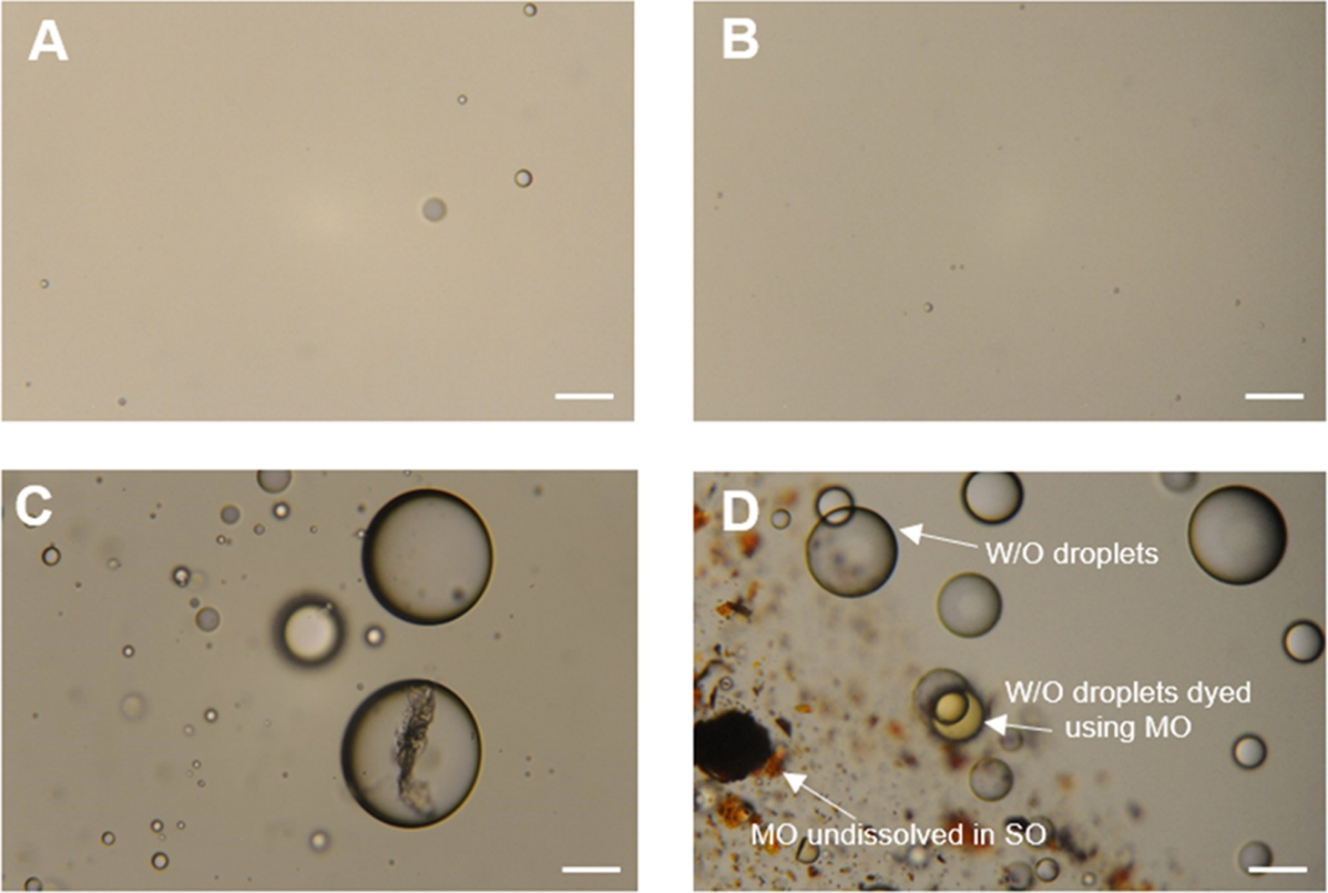
Representative microscopy images of W/O droplets in the porcine model (animal no. 931, SO viscosity 5000 mPa·s). (A) Aspiration through a 23-gauge needle, (B) aspiration through a 25-gauge needle and (C) direct extraction. (D) Methylene orange (MO) staining confirming the aqueous nature of the droplets in direct extraction sample. White scale bars = 100 μm.

A pronounced effect of the sampling method on the apparent droplet size distribution was observed. As illustrated by representative histograms from animal no. 931 for SO 5000 mPa·s (Figure 2), aspiration through different needle gauges resulted in marked shifts in droplet size distributions. Narrower needles were associated with a shift toward smaller detected droplets. However, W/O droplets were detected in samples obtained by direct extraction without a needle, indicating that droplets were already formed and did not arise during aspiration.

**Figure 2:**
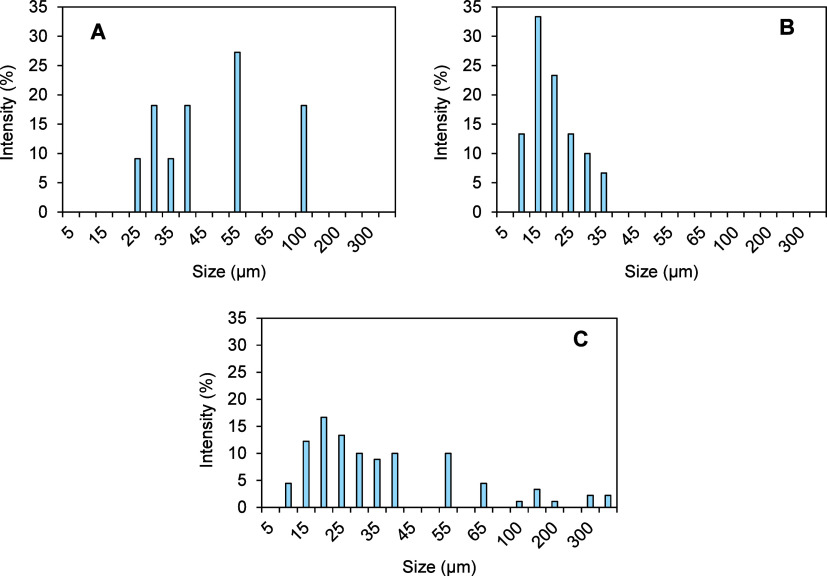
Droplet size distributions for representative samples from animal no. 931 (SO viscosity 5000 mPa·s) obtained by (A) 23-gauge aspiration, (B) 25-gauge aspiration and (C) direct extraction.

Comparison of samples obtained by direct extraction without needle aspiration revealed interindividual variability between animals and a viscosity-dependent shift in droplet size distributions (Figure 3). Higher SO viscosity qualitatively favored larger W/O droplets, consistent with reduced shear-induced fragmentation in highly viscous continuous phases.^33^ Direct extraction therefore eliminates aspiration-induced shear artifacts and reveals interindividual variability.

**Figure 3:**
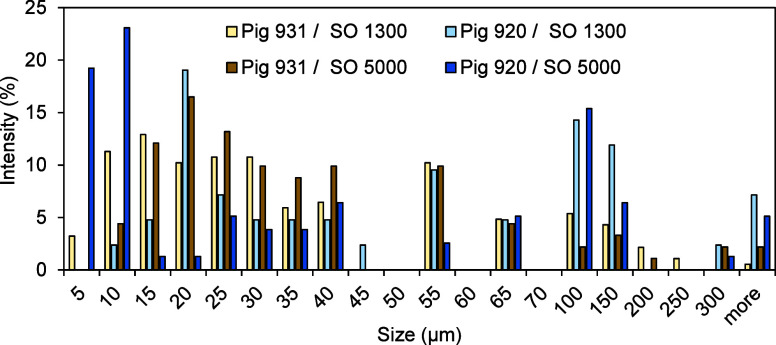
Comparison of W/O droplet size distributions obtained by direct extraction without needle aspiration from animals no. 920 and 931 for SO viscosities of 1300 and 5000 mPa·s.

Conversely, for each SO viscosity, the highest number of detected droplets (Table 1) was associated with a different sampling method, underscoring that viscosity alone does not determine the absolute number of detectable droplets. For SO with a viscosity of 1300 mPa·s, sampling through a 23-gauge needle resulted in the highest droplet counts in both animals, whereas aspiration through a 25-gauge needle led to the absence of detectable droplets. In contrast, for SO with a viscosity of 5000 mPa·s, the response to sampling was weaker and more heterogeneous: direct extraction yielded the highest droplet counts, aspiration through a 23-gauge needle resulted in a marked reduction, and 25-gauge aspiration showed variable outcomes between animals.

**Table 1:** Summary of W/O Droplet Counts Detected in Porcine Samples for Both Animals, Both SO Viscosities, and All Sampling Methods

	SO 1300 mPa·s		SO 5000 mPa·s	
aspiration method	Pig 920	Pig 931	Pig 920	Pig 931
direct	41	195	77	90
23-g	117	87	10	11
25-g	0	0	0	30

Taken together, these findings indicate that SO viscosity does not directly determine the presence or absence of W/O droplets but rather modulates the sensitivity of the system to shear-induced effects during sampling. Negative findings in individual samples should therefore not be interpreted as the absence of droplets in the eye but rather as a consequence of sampling-related bias and detection limits of optical microscopy.

Finally, quantitative analysis of intrasample variability (Table S2 in Supporting Information) confirmed that W/O droplet populations were highly polydisperse under all conditions, as reflected by standard deviations comparable to or exceeding the corresponding mean droplet diameters in several samples. Directly extracted samples generally exhibited larger mean droplet diameters and broader size distributions, whereas aspiration through 23-gauge or 25-gauge needles shifted the distributions toward smaller diameters and, in some cases, reduced variability, consistent with shear-induced droplet fragmentation. Image-to-image variability in droplet counts further revealed pronounced spatial heterogeneity within individual samples. Consistent with the trends observed in Table 1, no systematic viscosity-dependent trend in droplet counts was observed between 1300 mPa·s and SO 5000 mPa·s, further supporting that sampling-induced shear, rather than viscosity alone, is the dominant factor shaping the apparent droplet populations.

### 3.2 Human Silicone Oil Samples

SO samples were obtained from eight patients undergoing routine SO removal. When samples were collected with active intraocular infusion, W/O droplets were detected in all cases. The droplet size distributions were broad and heterogeneous, with both sub-50 μm and larger droplets observed, and significant variability between individual samples (Figures S4 and S5 in Supporting Information). These observations suggest that active infusion promotes mixing between SO and aqueous fluid during sampling, leading to the formation of artifactual W/O droplets.

In contrast, when SO was sampled without active infusion, W/O droplets were detected in only three of the eight patients (patients 2, 6 and 8). In the remaining five patients, no optically detectable droplets were observed in samples obtained without infusion. Similarly to the observations in the porcine model, the absence of droplets in these samples should not be interpreted as their absence in the eye. Conversely, the detection of W/O droplets in samples collected without infusion provides evidence that these droplets were present in vivo and did not arise as an artifact of sampling.

In patients 2 and 6 (Figure S5 in Supporting Information), W/O droplets were observed following aspiration through both 23-gauge and 25-gauge needles. Droplet size showed a clear qualitative dependence on needle diameter, with larger droplets detected after 23-gauge aspiration and smaller droplets after 25-gauge aspiration, consistent with shear-induced droplet fragmentation.

W/O droplets were detected in explanted SO samples from 3 out of 8 patients when sampling was performed without active infusion. Although the cohort size is limited this incidence (∼35%) is in the same order of magnitude as the reported clinical occurrence of O/W emulsification droplets in the anterior chamber after long-term SO tamponade, which has been reported in approximately 20% of cases in clinical studies.^34^ This qualitative correspondence suggests that the formation of W/O microstructures within the oil phase may be a clinically relevant but underrecognized counterpart to conventional O/W emulsification observed in the aqueous phase.

The observed variability between individual patients further indicates that W/O droplet formation is not uniform. Although the present study was not designed to identify clinical determinants, several factors that evolve over time may plausibly contribute to this variability. These include the duration of SO tamponade, which may influence the extent of interfacial aging and accumulation of surface-active species; patient-specific factors such as ocular motion and activity, which affect shear exposure; and the underlying pathology or inflammatory status, which may alter the composition of intraocular fluids and protein content.^35–37^

### 3.3 In Vitro Experiments Addressing Sampling-Induced Artifacts

To isolate the mechanical contribution of sampling to droplet size distributions and biological factors, complementary in vitro experiments were performed using preformed W/O emulsions. Aspiration was performed at a constant flow rate to allow controlled comparison between needle diameters.

The selected flow rate was not intended to replicate clinical aspiration conditions but to isolate the effect of needle diameter on droplet fragmentation. Flow rate is a key parameter governing shear forces during aspiration and thus directly influences droplet deformation and breakup. In the present study, it was intentionally kept constant to isolate the effect of geometric confinement. Consequently, the influence of flow rate on droplet fragmentation was not systematically investigated, and the observed droplet size distributions should be interpreted within this constrained experimental framework. Under clinical conditions, variations in aspiration rate are expected to modulate shear exposure and may therefore contribute to differences in droplet size distributions.

As shown in Figure 4 (representative microscopy images in Figure S6A–C in Supporting Information), aspiration of preformed W/O emulsions through needles resulted in a pronounced shift in droplet size distributions toward smaller sizes, demonstrating shear-induced droplet fragmentation during needle passage. Importantly, the emulsion was already present prior to aspiration, confirming that the observed changes reflect fragmentation rather than de novo droplet formation. Narrower needles produced a stronger shift toward smaller droplets, consistent with higher effective shear during aspiration.

**Figure 4:**
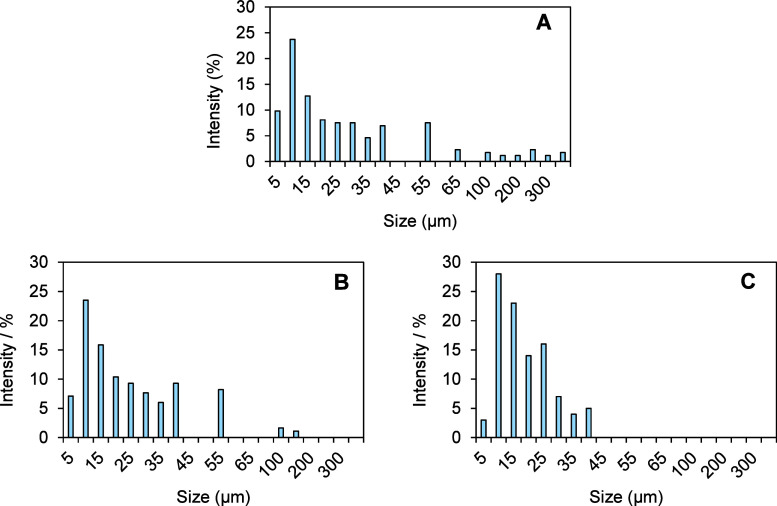
Droplet size distributions of model W/O emulsions before and after aspiration through needles of different gauge. (A) Preformed W/O emulsion prior to aspiration; (B) droplet size distribution after aspiration through a 23-gauge needle and (C) droplet size distribution after aspiration through a 25-gauge needle.

Droplet fragmentation during aspiration is consistent with classical emulsion breakup theory in shear flow. Droplet deformation and breakup are governed by the viscosity ratio and the capillary number (*Ca*; see eq 1), which reflects the balance between viscous shear stress and interfacial tension.^38^ In the presence of surface-active species, adsorption at the oil–water interface alters the interface, potentially giving rise to complex breakup modes. These include classical breakup modes such as binary breakup, satellite droplet formation via Rayleigh–Plateau instability, and tip streaming at high *Ca*;^39–41^ see Figure 5. To mechanistically interpret the observed needle-dependent changes in droplet size distributions, aspiration through narrow-gauge needles can be conceptualized as flow through a confined microcapillary shear reactor. Pre-existing W/O droplets suspended in SO enter a high-shear microcapillary environment, where hydrodynamic stresses induce droplet deformation and fragmentation. Given the low Reynolds number (see eq 2) regime characteristic of highly viscous SOs and submillimeter capillary dimensions (*Re* ≪ 1), the flow is laminar and dominated by viscous forces, resulting in strong shear gradients near the capillary wall.^42^ This framework explains the shift toward smaller droplet sizes with decreasing needle diameter and highlights that sampling itself can significantly alter apparent droplet size distributions.

1 $$Ca=\frac{\mu U}{\gamma }$$

2 $$Re=\frac{\rho UD}{\mu }$$

where ρ is the density of SO (kg·m^–3^), *U* the characteristic flow velocity (m·s^–1^), *D* the needle diameter (mm), μ the dynamic viscosity (Pa·s), and γ the interfacial tension (mN·m^–1^).

**Figure 5:**
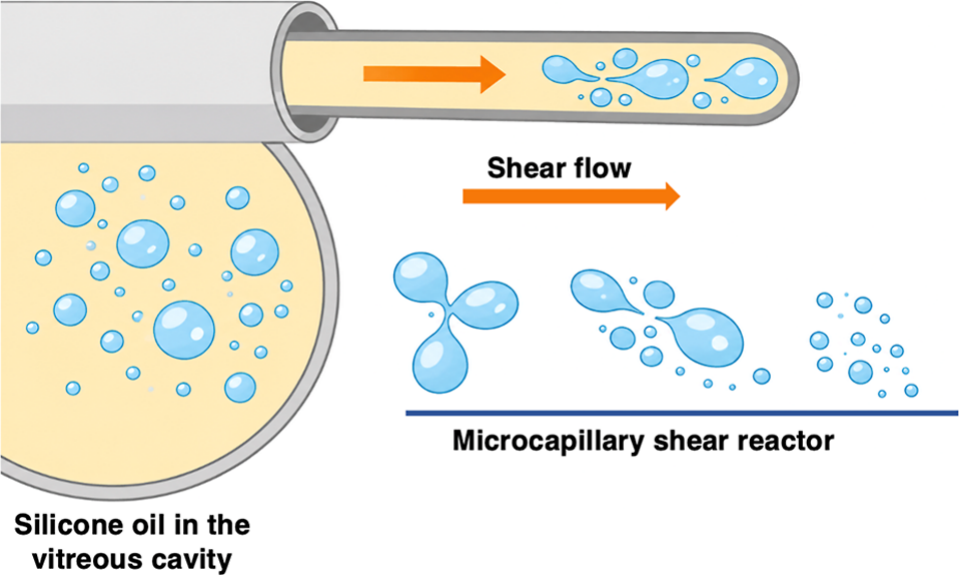
Schematically illustrates aspiration through a narrow-gauge needle as a microcapillary shear reactor.

To further quantify this interpretation, the relevant dimensionless numbers were estimated using order-of-magnitude analysis. Taking representative properties of SO (ρ ≈ 970 kg·m^–3^, μ = 1–5 Pa·s for both tested SOs), needle diameters of 0.5–0.6 mm, and clinically relevant flow rates on the order of 0.1–1 mL·s^–1^, the corresponding mean velocities are in the range *U* ≈ 0.3–5 m·s^–1^. Substitution yields Reynolds numbers *Re* ≈ 0.03–1.5, confirming predominantly laminar, viscous-dominated flow conditions. For interfacial tensions reduced by protein adsorption (γ ≈ 10–30 mN·m^–1^), the corresponding capillary numbers are *Ca* ≈ 10 to 10^3^, indicating that viscous stresses are sufficient to drive droplet deformation and breakup.

In addition to these dimensionless considerations, needle diameter itself is not the only parameter governing droplet behavior during aspiration. The associated pressure gradients and internal flow profiles are also expected to contribute to the observed differences between 23-gauge and 25-gauge aspiration. Under laminar capillary flow conditions, decreasing the needle diameter increases both the hydrodynamic resistance and the shear gradients experienced by droplets passing through the capillary.^43^ The present results should therefore not be interpreted as reflecting a purely geometric diameter effect, but rather as the consequence of a coupled hydrodynamic change in confinement, velocity profile, shear field, and pressure drop within the aspiration needle. In this sense, a smaller needle diameter creates a distinct capillary flow environment characterized by steeper shear gradients and increased pressure drop, which together promote droplet fragmentation.

The presented droplet size distributions are inherently limited by the optical detection threshold (∼1.1 μm), which excludes submicron droplets from analysis (see Section 3.5 for further discussion). Submicron droplets are a well-established feature of emulsified systems and may arise from shear-induced breakup.^44–46^ As a consequence, the reported size distributions likely underestimate the total droplet number and are skewed toward larger size fractions. Importantly, submicron droplets contribute disproportionately to the total interfacial area and can significantly affect physicochemical properties such as light scattering and interfacial transport, despite remaining undetected by optical microscopy.^46,47^

Control experiments further confirmed that needle passage of SO alone does not induce W/O droplet formation (Figure S6D in Supporting Information). To assess infusion-related artifacts, a simplified in vitro model experiment was performed in which an aqueous phase was continuously introduced into SO during sampling.

Under these conditions, W/O droplets were readily observed in the collected samples (representative images shown in Figure S6E in Supporting Information). The corresponding droplet size distribution (Figure S7 in Supporting Information) was broad, spanning from several micrometers to >100 μm, with a pronounced tail toward larger droplet sizes. It demonstrates that the copresence of aqueous inflow and oil aspiration can promote the formation of large W/O droplets under controlled conditions. These findings support the interpretation that infusion-based sampling may contribute to the generation or amplification of large droplets observed in human samples.

Together, these findings demonstrate that sampling can substantially alter the apparent droplet size distribution through both mechanical fragmentation and, under infusion-related conditions, the generation or amplification of W/O droplets. However, these processes do not represent the primary origin of W/O droplets detected in vivo.

### 3.4 Cross-Model Mechanistic Framework for W/O Droplet Formation in Intraocular Silicone Oil

The combined evaluation of porcine explants, human SO samples, and previously published in vitro data enables a cross-model comparison of W/O droplet formation mechanisms while highlighting the intrinsic limitations of each system. The aim is to assess convergence at the level of emulsion type, droplet size range, and underlying mechanisms rather than quantitative equivalence between models. Instead, the consistency between in vitro, porcine, and human observations supports a unified mechanistic interpretation of W/O droplet formation across experimental systems.

Our previous in vitro study^18^ demonstrated that, under protein-rich conditions and high oil fractions, SO readily forms stable W/O emulsions when exposed to mechanical shear. These emulsions exhibited droplet sizes ranging from tens to hundreds of micrometers. The present porcine data show a close qualitative correspondence: W/O droplets were detected directly in SO explanted after tamponade, with droplet sizes falling within the same order of magnitude, particularly when sampling conditions minimized shear-induced fragmentation.

However, important biological differences between porcine and human eyes must be considered. In pigs, a substantially larger amount of residual vitreous typically remains after vitrectomy compared to humans. In our earlier work,^18^ we demonstrated that liquefied vitreous residue can itself act as a source of W/O droplets when dispersed in SO under mechanical agitation. The higher vitreous residue in porcine eyes therefore represents an intrinsic factor that may increase the likelihood of W/O droplet formation, independent of other variables. Consequently, the porcine system should be regarded as a mechanistically informative but biologically permissive model that may amplify phenomena occurring more subtly in humans.

In addition to compositional differences, variability in ocular motion and postoperative behavior may also influence the effective shear exposure within the vitreous cavity, thereby contributing to differences in droplet formation between experimental systems.

In particular, pigs exhibit substantially higher postoperative activity compared to human patients, resulting in increased mechanical loading. Given the shear-dependent nature of emulsification demonstrated both in vitro and in vivo, this increased mechanical loading likely contributes to enhanced W/O droplet formation in the porcine model.

Human explant data provide an intermediate perspective. W/O droplets were detected only in a subset of patients and exclusively in samples obtained without infusion, indicating that their presence cannot be attributed to a systematic sampling artifact. However, the limited cohort size (further discussion in Section 3.5) precludes robust identification of clinical factors associated with W/O droplet occurrence. Although the atypical cases shared specific perioperative or postoperative circumstances, the available documentation does not allow definitive attribution of causality.

At the physicochemical level, previous works demonstrated that interfacial proteins exert a dual role in SO-aqueous systems. Proteins are amphiphilic macromolecules that (1) adsorb at oil–water interfaces and reduce interfacial tension, thereby facilitating droplet formation and stabilization, while also promoting the development of structured interfaces that stabilize W/O droplets against further breakup.^48,49^ Phase behavior depends on composition and phase ratio, with coexistence of W/O and O/W emulsions under balanced conditions and selective formation under asymmetric compositions^18^ (summarized in Table S1 in the Supporting Information). It indicates that droplet formation in SO is governed not by a single parameter, but by the combined effects of composition, phase ratio, and mechanical energy input.

While direct measurements of equilibrium interfacial tension were not performed in the present study, this effect is well established in the literature^49,53–57^ (detailed in Supporting Information). Furthermore, in biological systems, the interface is dynamically covered by proteins and exhibits viscoelastic properties, rendering equilibrium interfacial tension insufficient to describe droplet behavior.^50–52^ For this reason, (2) interfacial viscoelasticity represents a more appropriate descriptor of interfacial mechanics under biologically relevant conditions.

Therefore, rheological measurements reported in our previous study^18^ provide direct quantitative insight into the viscoelastic properties governing droplet formation and stability in SO-aqueous systems (for experimental details, see original ref 18 and Supporting Information). Interfacial viscoelastic measurements revealed that, in the absence of proteins (distilled water or saline solution), the interface exhibits negligible mechanical strength, with near-zero complex modulus and predominantly viscous behavior. In contrast, biologically relevant aqueous media (model aqueous humor) induce the formation of a viscoelastic interfacial film characterized by a high complex modulus and a mixed elastic-viscous response.

Importantly, the interfacial response depends not only on protein adsorption but also on the viscosity of the oil phase. While both SO with viscosity 1000 and 5000 mPa·s exhibit comparable magnitudes of interfacial modulus, the higher-viscosity oil shows a more dissipative interfacial response, as reflected by increased phase shift values. This indicates that interfacial dynamics are governed by a balance between elastic energy storage within the protein film and viscous dissipation within the oil phase.

In the context of the present study, these rheological characteristics provide a mechanistic basis for the observed in vivo formation and persistence of W/O droplets. Under shear conditions generated by ocular motion, protein-covered interfaces—shown in previous measurement^18^ to form viscoelastic interfacial films—enable droplet formation by lowering interfacial tension while simultaneously stabilizing the resulting structures through viscoelastic resistance. During aspiration, these droplets are subjected to intense, spatially confined shear fields, where their deformation and breakup are controlled by the interplay between viscous stresses and interfacial viscoelasticity.

This framework explains why droplet fragmentation during needle passage depends not only on geometric confinement but also on the rheological properties of the system, and why higher viscosity alone does not prevent emulsification. Instead, droplet behavior emerges from the coupled dynamics of bulk viscosity and interfacial rheology, highlighting the importance of protein-mediated interfaces in governing multiphase behavior in intraocular SO.

Cross-model comparison further demonstrated that W/O droplet size distributions overlapped across porcine, human, and in vitro aspiration systems, with both small (<20 μm) and large (>100 μm) droplets detected (Figure 6B). The in vitro aspiration experiment reproduced key features of the in vivo distributions, supporting the interpretation that needle aspiration acts as a confined microcapillary shear process that fragments pre-existing droplets. Human samples exhibited droplet size distributions within the range observed in the porcine model, supporting the translational relevance of the porcine system and confirming that W/O droplet formation is not species-specific. Similarly, W/O droplet size distributions obtained by direct extraction from porcine eyes were qualitatively consistent with those generated in vitro under biologically relevant conditions (Figure 6A). Interindividual variability between animals was observed, reflecting biological heterogeneity in interfacial composition and mechanical exposure, but the overall size ranges and distribution shapes were comparable across models.

**Figure 6:**
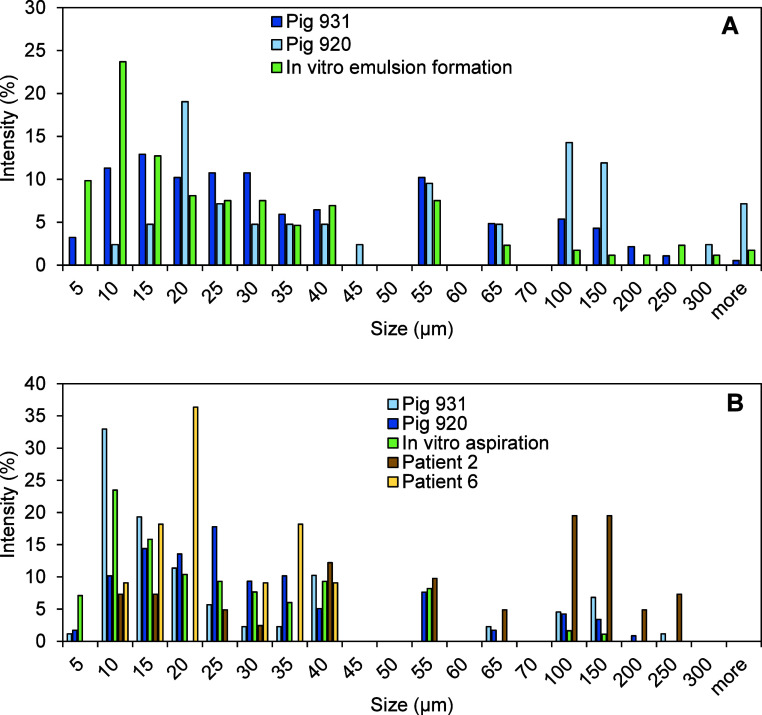
Cross-model comparison of W/O droplet size distributions. (A) Comparison of W/O droplet size distributions obtained by direct extraction from both porcine eyes (SO viscosity 1300 mPa·s) and W/O droplets formed in vitro under biologically relevant conditions as reported previously. (B) Comparison of W/O droplet size distributions for both porcine eyes, human patients 2 and 6, and in vitro aspiration through a 23-gauge needle for SO 1300 mPa·s (for the human sample used SO 5000 mPa·s).

Taken together, the three experimental levels—in vitro systems, porcine explants, and human samples—form a complementary hierarchy of evidence. In vitro models provide controlled mechanistic insight into shear-driven emulsification and interfacial stabilization. The porcine model demonstrates that the same processes occur in a living eye under conditions of enhanced mechanical stress and biological complexity. Human explants confirm the clinical relevance of W/O droplet formation while highlighting interindividual variability. Collectively, these data support a unified mechanistic framework in which W/O droplets arise as a consequence of shear-driven emulsification in oil-dominated, protein-containing environments, modulated by biological context and sampling conditions rather than by a single dominant clinical factor.

Finally, Figure 7 schematically summarizes the conceptual link between in vivo observations and in vitro validation. The left part of the scheme reflects the in vivo findings, where W/O droplets are detected in porcine and human SO samples, with their apparent size and abundance strongly influenced by the sampling method. The right part represents the corresponding in vitro systems, which reproduce both the formation of W/O droplets under controlled shear conditions and their subsequent modification during sampling, including shear-induced fragmentation during aspiration and artifactual droplet generation under infusion-like conditions. The bottom panel integrates these observations into a unified mechanistic framework, in which W/O droplets are formed in vivo as a result of shear-driven emulsification in protein-containing systems and are subsequently modified during sampling through microcapillary shear and mixing effects.

**Figure 7:**
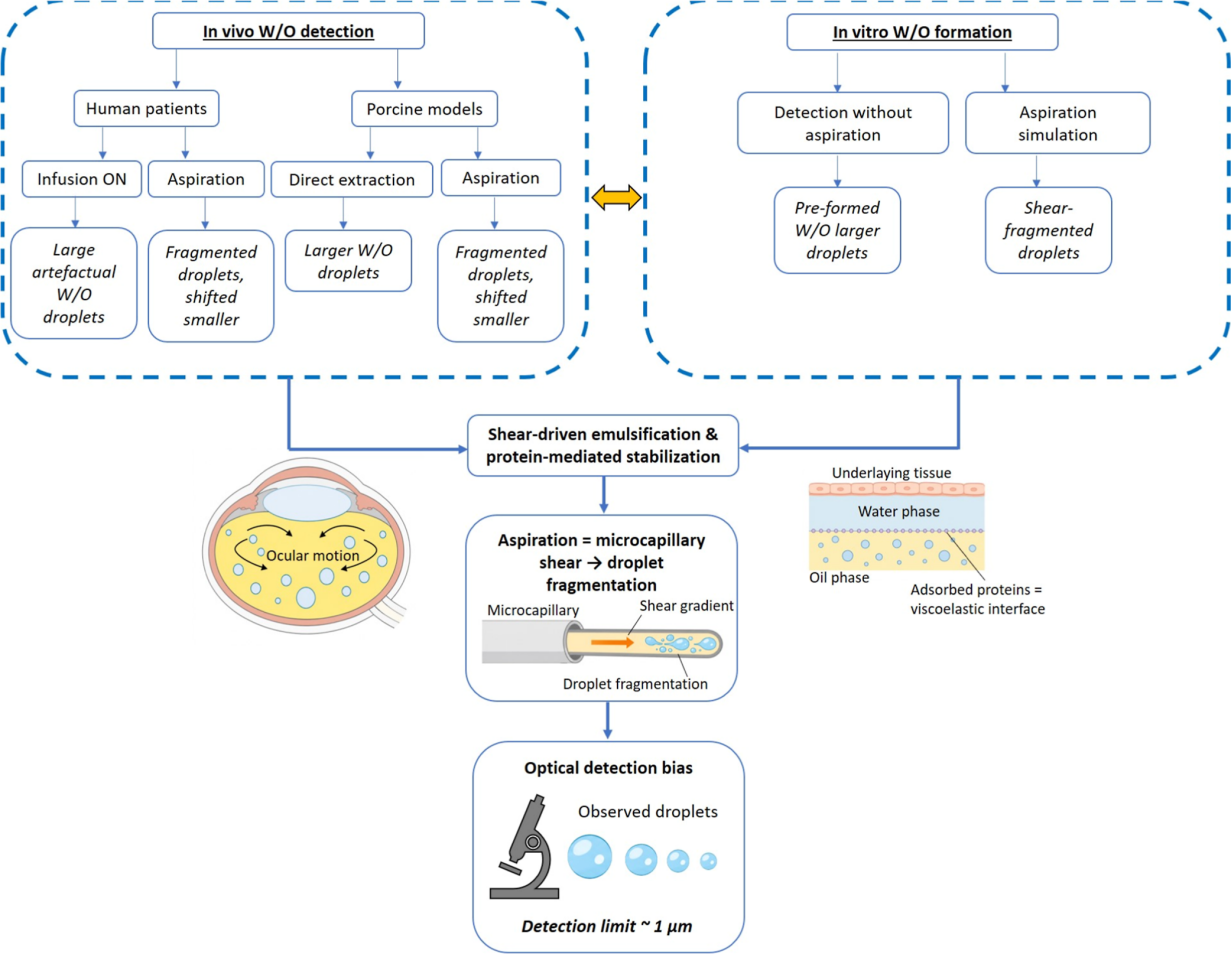
Integrated schematic overview of the experimental evidence and proposed mechanistic framework.

### 3.5 Potential Implications and Study Limitations

To date, no studies have directly investigated the biological effects of W/O droplets within the SO phase during long-term vitreous tamponade. Most available clinical and experimental evidence relates to conventional O/W emulsification, which has been associated with inflammatory responses, elevated intraocular pressure, and interactions with ocular tissues.

In this context, the potential biological relevance of W/O droplets remains largely unexplored. However, their presence introduces additional internal interfaces within the SO phase, which may act as compartments for dissolved proteins, ions, or inflammatory mediators. Such compartmentalization could influence local microenvironments and potentially contribute to interfacial activity or transport processes within the tamponade.

From a physicochemical perspective, the presence of dispersed aqueous domains may also affect the effective optical and mechanical properties of SO. Submicron and micron-sized droplets are known to contribute to light scattering,^30^ which could influence visual quality, while larger droplets may alter the rheological behavior or stability of the tamponade under dynamic conditions. Although these considerations are consistent with established principles of multiphase systems and emulsion behavior, direct experimental or clinical evidence linking W/O droplets to specific biological outcomes is currently lacking.

Based on the combined in vitro and in vivo observations, a dynamic mechanism can be proposed. W/O droplets are likely formed through repeated shear events within the vitreous cavity, such as those induced by ocular motion, particularly in the presence of protein-containing aqueous phases. At early stages, droplet formation may be limited by the availability of interfacially active species. Over time, adsorption of proteins and other surface-active components at the oil–water interface may lead to interfacial aging, resulting in increased interfacial viscoelasticity and enhanced stabilization of dispersed droplets. In this context, droplet populations are expected to evolve dynamically, with ongoing formation, fragmentation, and stabilization processes occurring throughout the tamponade period.

While the biological implications of W/O droplets and their dynamic evolution remain to be elucidated, these findings already have direct practical relevance for the analysis and interpretation of explanted SO samples. From a practical perspective, the present findings highlight the need for careful standardization of sampling procedures to ensure reproducibility and comparability across different clinical settings. Based on the combined in vivo and in vitro observations, several key recommendations can be formulated:

(i) Sampling should be performed with the intraocular infusion turned off to avoid coaspiration of aqueous fluid, which was shown to induce the formation of large, artifactual W/O droplets.
(ii) The use of narrow-gauge needles should be carefully considered, as aspiration through smaller diameters introduces higher shear stresses that can lead to droplet fragmentation and shifts in the observed size distribution. Where possible, direct extraction without capillary constriction provides the most representative assessment of in vivo droplet populations.
(iii) Sampling procedures should be performed under controlled and reproducible conditions, including consistent aspiration rates and minimal mechanical perturbation during handling.
(iv) The interpretation of droplet size distributions should explicitly account for method-dependent artifacts, particularly when comparing results obtained using different sampling strategies or instrumentation.

Further studies combining detailed physicochemical characterization with clinical observations will be required to clarify the biological relevance of W/O droplets, including their time-dependent evolution, which requires longitudinal sampling and was beyond the scope of the present study, while the present findings already provide a framework for improving reproducibility and minimizing sampling-related variability in future studies of explanted SO.

Finally, several limitations of this work should be acknowledged:

(i) The number of animals and human samples included in this study was limited, which precludes statistical analysis and population-level generalization. The present work was therefore not designed to provide quantitative estimates of prevalence or to identify clinical risk factors associated with W/O droplet formation. Human explant samples are currently being collected in an ongoing prospective study to enable systematic evaluation in a larger clinical cohort, whereas the number of animals was intentionally minimized in accordance with ethical guidelines for animal research.

Instead, the primary aim was to establish the existence of W/O droplets in vivo and to identify the underlying physicochemical mechanisms governing their formation and behavior. In this context, the combined use of controlled in vitro experiments, a porcine in vivo model, and human explant analysis provides a consistent, cross-model mechanistic framework rather than a statistically powered clinical data set.

(ii) In addition, each sampling condition was applied only once per eye, and the collected samples therefore do not constitute independent biological replicates for statistical comparison. Repeated sampling from the same eye under identical conditions was not feasible without further perturbing the system and potentially altering the droplet population. Accordingly, the presented distributions should be interpreted as representative, condition-specific observations within a mechanistic framework rather than as statistically replicated measurements.
(iii) The present optical microscopy-based analysis is the detection threshold of approximately 1.1 μm, below which droplets cannot be reliably resolved. Consequently, submicron W/O droplets may be present but remain undetected, potentially leading to an underestimation of total droplet counts and a bias toward larger droplet sizes. This is particularly relevant for samples subjected to high shear, where fragmentation is expected to generate smaller droplets. Importantly, the absence of optically detectable droplets should therefore not be interpreted as the absence of dispersed aqueous domains within the SO phase.

Submicron droplet populations are well documented in emulsion systems and can significantly influence light scattering and optical properties despite their limited contribution to volume-based size distributions.^58^

While complementary techniques such as dynamic light scattering (DLS) could, in principle, provide information on submicron droplet populations, their application in the present system is limited by several well-recognized factors.^59^ In particular, DLS measurements are inherently biased toward larger scatterers due to the intensity-weighted nature of the signal (approximately proportional to the sixth power of particle diameter), and show limited resolving power in highly polydisperse or multimodal systems, where smaller populations may be masked or inaccurately represented.^60–62^

In contrast, optical microscopy enables direct visualization of droplet morphology and phase identification (e.g., via selective staining), which is essential for confirming the presence of W/O structures. For these reasons, optical microscopy was selected as the primary characterization method in this study.

(iv) Biological differences between the porcine model and the human eye, including residual vitreous volume and postoperative activity, may influence the extent of emulsification and limit direct quantitative extrapolation. In particular, differences in behavioral patterns and postoperative compliance may result in varying levels of ocular motion and shear exposure, which are expected to directly affect droplet formation and fragmentation processes.
(v) In confined capillary systems, flow rate, pressure gradient, and geometric confinement are intrinsically coupled and cannot be independently varied. Consequently, the observed droplet size distributions reflect combined hydrodynamic effects rather than independently controlled variables. This limits the ability to directly translate the experimental findings to clinical aspiration conditions, where flow rates and pressure gradients may vary between procedures and operators.

Despite these limitations, the consistent detection of W/O droplets across experimental levels supports a unified mechanistic framework in which shear-driven emulsification in oil-dominated, protein-containing environments leads to droplet formation during SO tamponade. Future work combining larger clinical cohorts, controlled in vitro studies, and functional assessments will be essential to clarify the clinical relevance of this phenomenon and its potential impact on patient outcomes.

## 4 Conclusions

In this work, we provide direct in vivo evidence for the formation of W/O droplets during long-term vitreous tamponade, demonstrating that intraocular SO functions as a dynamic multiphase soft material rather than a single continuous phase. Using a porcine model and explanted human samples, we show that W/O droplets are present within the oil phase and that their observed size distributions are strongly influenced by sampling conditions. Aspiration through narrow-gauge needles induces shear-driven droplet fragmentation, whereas concurrent aqueous inflow can generate large artifactual droplets, highlighting clinical explantation as a confined microcapillary shear process that can bias ex vivo microstructural characterization.

By integrating in vivo observations with in vitro phase behavior and interfacial viscoelasticity data, we establish a mechanistic framework in which shear stresses and protein-mediated interfacial stabilization govern W/O droplet formation, persistence, and fragmentation in oil-dominated biological environments. These findings reinterpret intraocular SO as a confined, protein-active multiphase biomaterial system and underscore the importance of interfacial phenomena in determining its long-term behavior.

Beyond ophthalmology, this study illustrates that sampling and handling procedures can introduce shear-induced microstructural artifacts in viscous multiphase biomaterials. Our results therefore provide a broader framework for interpreting droplet microstructures in complex biological systems and for designing future studies and clinical protocols that minimize mechanically induced perturbations.

## Supplementary Material



## Notes

Detailed description of the image analysis workflow for droplet size quantification; schematic illustration of the sampling and imaging strategy; quantitative analysis of intrasample technical variability in porcine samples; complete droplet size distributions for porcine and human samples; representative microscopy images of W/O droplets in human explants; additional droplet size distributions for patients with and without infusion; and control in vitro experiments addressing sampling-induced artifacts, including needle-induced fragmentation and infusion-mimicking experiments (PDF)

## References

[1] Pal R. (2011). Rheology of
Simple and Multiple Emulsions. Curr. Opin. Colloid
Interface Sci..

[2] Li X., Xu X., Song L., Bi A., Wu C., Ma Y., Du M., Zhu B. (2020). High Internal
Phase Emulsion for Food-Grade 3D Printing
Materials. ACS Appl. Mater. Interfaces.

[3] Sjöblom, J. Emulsions and Emulsion Stability; Taylor & Francis, 2015.

[4] Tadros T. F. (1994). Fundamental
Principles of Emulsion Applications. Colloids
Surf., A.

[5] Yu J., Zong Y., Jiang C., Zhu H., Deng G., Xu G. (2020). Silicone Oil Emulsification after
Vitrectomy for Rhegmatogenous Retinal
Detachment. J. Ophthalmol..

[6] Dhoot A. S., Popovic M. M., Nichani P. A. H., Eshtiaghi A., Mihalache A., Sayal A. P., Yu H., Wykoff C. C., Kertes P. J., Muni R. H. (2022). Pars Plana Vitrectomy versus Scleral
Buckle: A Comprehensive Meta-Analysis of 15,947 Eyes. Survey of Ophthalmology.

[7] Ichhpujani P., Jindal A., Jay Katz L. (2009). Silicone Oil
Induced Glaucoma: A
Review. Graefes Arch. Clin. Exp. Ophthalmol..

[8] Cibis P. A., Becker B., Okun E., Canaan S., Louis S. (1962). The Use of
Liquid Silicone in Retinal Detachment Surgery. Arch. Ophthalmol..

[9] Patel A. V., Papakostas T. D., Elliot D. (2015). Silicone Oil Emulsification in Retina
Surgery. Retina Today.

[10] Chan Y. K., Ng C. O., Knox P. C., Garvey M. J., Williams R. L., Wong D. (2011). Emulsification of Silicone Oil and Eye Movements. Invest. Ophthalmol. Vis. Sci..

[11] Caramoy A., Hagedorn N., Fauser S., Kugler W., Groß T., Kirchhof B. (2011). Development of Emulsification-Resistant Silicone Oils:
Can We Go beyond 2000 MPas Silicone Oil?. Invest.
Ophthalmol. Vis. Sci..

[12] Riedel K. G., Gabel V.-P., Neubauer L., Kampik A., Lund O.-E. (1990). Intravitreal
Silicone Oil Injection: Complications and Treatment of 415 Consecutive
Patients. Graefes Arch. Clin. Exp. Ophthalmol..

[13] Toklu Y., Cakmak H. B., Ergun Ş. B., Yorgun M. A., Simsek Ş. (2012). Time
Course of Silicone Oil Emulsification. Retina.

[14] Savion N., Alhalel A., Treister G., Bartov E. (1996). Role of Blood Components
in Ocular Silicone Oil Emulsification Studies on an In Vitro Model. Invest. Ophthalmol. Vis. Sci..

[15] Nepita I., Repetto R., Pralits J. O., Romano M. R., Ravera F., Santini E., Liggieri L. (2020). The Role of
Endogenous Proteins on
the Emulsification of Silicone Oils Used in Vitreoretinal Surgery. BioMed Res. Int..

[16] Jaklová N., Kamenická B. (2026). In Vitro Silicone
Oil Emulsification in Retinal Detachment
Treatment: Methods, Designs, and Future Strategies. Acta Biomater..

[17] Williams R. L., Day M., Garvey M. J., English R., Wong D. (2010). Increasing the Extensional
Viscosity of Silicone Oil Reduces the Tendency for Emulsification. Retina.

[18] Kamenická B., Pěnkavová V., Lyko Vachková E., Orvalho S., Zedníková M., Jaklová N., Stavárek P., Reháčková M., Rajs P., Veith M., Klusoň P. (2026). Emulsification
Complexity of Silicone Oil in Retinal Surgery: In Vitro Insights into
Phase Behavior. ACS Omega.

[19] Ravera F., Dziza K., Santini E., Cristofolini L., Liggieri L. (2021). Emulsification and Emulsion Stability: The Role of
the Interfacial Properties. Adv. Colloid Interface
Sci..

[20] Miller J. B., Papakostas T. D., Vavvas D. G. (2014). Complications of Emulsified Silicone
Oil after Retinal Detachment Repair. Semin.
Ophthalm..

[21] Lu Y., Chan Y. K., Lau L. H., Wong D., Wong J. K. W., Shih K. C., Lai S. M., Shum H. C. (2020). Amphiphilic Additives
in Silicone Oil Tamponade and Emulsification: An Eye-on-a-Chip Study. Acta Ophthalmol..

[22] Lee M. C., Tan C., Ravanfar R., Abbaspourrad A. (2019). Ultrastable Water-in-Oil High Internal
Phase Emulsions Featuring Interfacial and Biphasic Network Stabilization. ACS Appl. Mater. Interfaces.

[23] Peters F., Arabali D. (2013). Interfacial Tension between Oil and
Water Measured
with a Modified Contour Method. Colloids Surf.,
A.

[24] Hammer M., Schickhardt S., Munro D. J., Scheuerle A., Mayer C. S., Auffarth G. U. (2022). Physicochemical
Properties of Explanted
Silicone Oil After Use as an Intraocular Tamponade. Transl. Vis. Sci. Technol..

[25] Pastor
Jimeno J. C., de la Rúa E. R., Fernández Martínez I., del Nozal Nalda M. J., Jonas J. B. (2007). Lipophilic Substances in Intraocular
Silicone Oil. Am. J. Ophthalmol..

[26] Brunner S., Izay B., Weidinger B., Maichel B., Binder S. (2011). Chemical Impurities
and Contaminants in Different Silicone Oils in Human Eyes before and
after Prolonged Use. Graefes Arch. Clin. Exp.
Ophthalmol..

[27] Pastor J. C., Del Nozal M. J., Marinero P., Díez O. (2006). Concentraciones
de Colesterol, Alfa -Tocoferol y Retinoides En Aceite de Silicona
Tras Su Utilizaciσn Como Sustitutivo Vνtreo. Arch. Soc. Esp. Oftalmol..

[28] Refojo M. F., Leong F.-L., Chung H., Ueno N., Nemiroff B., Tolentino F. I. (1988). Extraction
of Retinol and Cholesterol by Intraocular
Silicone Oils. Ophthalmology.

[29] Lakits A., Nennadal T., Scholda C., Knaus S., Gruber H. (1999). Chemical Stability
of Silicone Oil in the Human Eye after Prolonged Clinical Use. Ophthalmology.

[30] Hammer M., Britz L., Schickhardt S., Munro D., Khoramnia R., Scheuerle A., Mayer C. S., Uhl P., Łabuz G., Auffarth G. U. (2024). Straylight of Explanted Silicone Oil Samples to Predict
Emulsification. Ophthalmo. Sci..

[31] Schramke S., Schubert R., Frank F., Wirsig M., Fels M., Kemper N., Schuldenzucker V., Reilmann R. (2015). The Libechov Minipig
as a Large Animal Model for Preclinical Research in Huntington’s
Disease – Thoughts and Perspectives. Cesk. Neurol. Neurochir..

[32] Schneider C. A., Rasband W. S., Eliceiri K. W. (2012). NIH Image to ImageJ: 25 Years of
Image Analysis. Nat. Methods.

[33] Farsoiya P. K., Liu Z., Daiss A., Fox R. O., Deike L. (2023). Role of Viscosity in
Turbulent Drop Break-Up. J. Fluid Mech..

[34] Łątkowska M., Gajdzis M., Kaczmarek R. (2024). Emulsification of Silicone Oils:
Altering Factors and Possible Complications-A Narrative Review. J. Clin. Med..

[35] Murthy K. R., Goel R., Subbannayya Y., Jacob H. K., Murthy P. R., Manda S. S., Patil A. H., Sharma R., Sahasrabuddhe N. A., Parashar A., Nair B. G., Krishna V., Prasad T. K., Gowda H., Pandey A. (2014). Proteomic
Analysis of Human Vitreous
Humor. Clin. Proteomics.

[36] Bergfreund J., Bertsch P., Fischer P. (2021). Adsorption of Proteins to Fluid Interfaces:
Role of the Hydrophobic Subphase. J. Colloid
Interface Sci..

[37] Silva A. F., Pimenta F., Alves M. A., Oliveira M. S. N. (2020). Flow Dynamics
of Vitreous Humour during Saccadic Eye Movements. J. Mech. Behav. Biomed. Mater..

[38] Janssen J. J. M., Boon A., Agterof W. G. M. (1994). Droplet
Break-up in Simple Shear
Flow in the Presence of Emulsifiers. Colloids
Surf., A.

[39] Herrada M. A., Ponce-Torres A., Rubio M., Eggers J., Montanero J. M. (2022). Stability
and Tip Streaming of a Surfactant-Loaded Drop in an Extensional Flow.
Influence of Surface Viscosity. J. Fluid Mech..

[40] Stone H. A., Leal L. G. (1990). The Effects of Surfactants on Drop Deformation and
Breakup. J. Fluid Mech..

[41] Montanero J. M., Gañán-Calvo A. M. (2020). Dripping, jetting
and tip streaming. Rep. Prog. Phys..

[42] Squires T. M., Quake S. R. (2005). Microfluidics: Fluid Physics at the
Nanoliter Scale. Rev. Mod. Phys..

[43] Müller S. J., Mirzahossein E., Iftekhar E. N., Bächer C., Schrüfer S., Schubert D. W., Fabry B., Gekle S. (2020). Flow and Hydrodynamic
Shear Stress inside a Printing Needle during Biofabrication. PLoS One.

[44] Tadros, T. F. In Emulsions, Foams, Suspensions, and Aerosols; Schramm, L. L. , Ed.; Wiley, 2014

[45] Grace H. P. (1982). DISPERSION
PHENOMENA IN HIGH VISCOSITY IMMISCIBLE FLUID SYSTEMS AND APPLICATION
OF STATIC MIXERS AS DISPERSION DEVICES IN SUCH SYSTEMS. Chem. Eng. Commun..

[46] Mason T. G., Bibette J., Weitz D. A. (1996). Yielding and Flow
of Monodisperse
Emulsions. J. Colloid Interface Sci..

[47] Pal R. (2011). Rheology of
Simple and Multiple Emulsions. Curr. Opin. Colloid
Interface Sci..

[48] Chrysanthou A., Kanso H., Zhong W., Shang L., Gautrot J. E. (2023). Supercharged
Protein Nanosheets for Cell Expansion on Bioemulsions. ACS Appl. Mater. Interfaces.

[49] Nakamura K., Refojo M. F., Crabrree D. V. (1990). Factors Contributing to the Emulsification
of Intraocular Silicone and Fluorosilicone Oils. Invest Ophthalmol. Vis. Sci..

[50] Dickinson E. (2010). Food Emulsions
and Foams: Stabilization by Particles. Curr.
Opin. Colloid Interface Sci..

[51] Sagis L. M. C. (2011). Dynamic
Properties of Interfaces in Soft Matter: Experiments and Theory. Rev. Mod. Phys..

[52] Fuller G. G., Vermant J. (2012). Complex Fluid-Fluid Interfaces: Rheology
and Structure. Annu. Rev. Chem. Biomol. Eng..

[53] Beverung C. J., Radke C. J., Blanch H. W. (1999). Protein
Adsorption at the Oil/Water
Interface: Characterization of Adsorption Kinetics by Dynamic Interfacial
Tension Measurements. Biophys. Chem..

[54] Sah H., Choi S.-K., Choi H.-G., Yong C.-S. (2002). Relation of Dynamic
Changes in Interfacial Tension to Protein Destabilization upon Emulsification. Arch. Pharm. Res..

[55] Bergfreund J., Bertsch P., Fischer P. (2021). Adsorption of Proteins to Fluid Interfaces:
Role of the Hydrophobic Subphase. J. Colloid
Interface Sci..

[56] Lin L.-H., Bergfreund J., Fischer P., Bertsch P. (2025). Plant Protein Adsorption
at Oil–Water Interfaces: A Mapping Review Using Alternate Subphase
Tensiometry. Curr. Opin. Colloid Interface Sci..

[57] Baldursdottir S. G., Fullerton M. S., Nielsen S. H., Jorgensen L. (2010). Adsorption
of Proteins at the Oil/Water Interface-Observation of Protein Adsorption
by Interfacial Shear Stress Measurements. Colloids
Surf., B.

[58] Chantrapornchai W., Clydesdale F., McClements D. J. (1998). Influence of Droplet Size and Concentration
on the Color of Oil-in-Water Emulsions. J. Agric.
Food Chem..

[59] Frisken B.
J. (2001). Revisiting
the Method of Cumulants for the Analysis of Dynamic Light-Scattering
Data. Appl. Opt..

[60] Carpenter D. K. (1977). Dynamic
Light Scattering with Applications to Chemistry, Biology, and Physics
(Berne, Bruce J.; Pecora, Robert). J. Chem.
Educ..

[61] Stetefeld J., McKenna S. A., Patel T. R. (2016). Dynamic
Light Scattering: A Practical
Guide and Applications in Biomedical Sciences. Biophys. Rev..

[62] Pecora R. (2000). Dynamic Light
Scattering Measurement of Nanometer Particles in Liquids. J. Nanopart. Res..

