# Supplementary material for: When Shear and Interfaces Matter: In Vivo Water-in-Silicone Oil Droplet Formation during Long-Term Vitreous Tamponade
Source: ACS Omega. 2026 Jun 1;11(23):34429–41. doi: 10.1021/acsomega.6c02409 (PMC13281013; doi:10.1021/acsomega.6c02409)
Supplement: Supplementary file 1 [file ao6c02409_si_001.pdf]

When Shear and Interfaces Matter: In Vivo Water-in-Silicone Oil Droplet Formation during Long-Term Vitreous Tamponade

Miroslav Veith [a,b], *Monika Reháčková* [a,b], *Patrik Rajs* [a,b], *Jan Motlík* [c], *Barbora Kamenická* [d,e*] *and Petr Klusoň* [d,e]

[a] Third Faculty of Medicine, Charles University, Ruská 87, 100 00 Prague, Czech Republic

[b] Department of Ophthalmology, University Hospital Kralovské Vinohrady and Third Faculty of Medicine, Charles University, Šrobárova 50, 100 34 Prague

[c] Institute of Animal Physiology and Genetics, Academy of Sciences of the Czech Republic, Rumburská 89, 277 21 Liběchov, Czech Republic

[d] Institute of Chemical Process Fundamentals of the Czech Academy of Sciences, Rozvojová 2/135, 165 00 Prague, Czech Republic

[e] Institute for Environmental Studies, Faculty of Science, Charles University, Benátská 2, 110 00 Prague, Czech Republic

Supporting Information

Supporting Text

Image analysis workflow: Droplet size and distribution were determined using ImageJ software (version 1.54g, National Institutes of Health, USA). Images were first calibrated using the embedded scale bar via the "Set Scale" function to ensure accurate size measurements. Color images were analyzed using the "Color Threshold" tool to segment droplets from the background based on hue, saturation, and brightness (HSB) parameters. Thresholding was performed to achieve optimal separation between droplets and background, and the selected settings were applied consistently within each dataset. Following segmentation, droplet analysis was performed using the "Analyze Particles" function, which provided the number of detected droplets and their projected areas. Basic filtering was applied to exclude obvious background artefacts and non-droplet features. Assuming spherical symmetry in 2D projection, the equivalent droplet diameter was calculated from the measured area. The resulting data were used to construct droplet size distributions. For each sample, multiple droplets were deposited on the microscope slide and multiple images were acquired, allowing representative characterization of the droplet population and reducing bias associated with local heterogeneity. The analysis workflow followed a consistent procedure across all datasets to ensure comparability of the resulting size distributions.

Protein-mediated reduction of oil–water interfacial tension: Proteins are well-established surface-active amphiphilic macromolecules that adsorb at oil–water interfaces and reduce interfacial tension through adsorption and conformational rearrangements. Dynamic interfacial tension measurements using pendant drop tensiometry have demonstrated that protein adsorption kinetics directly correlate with a time-dependent decrease in interfacial tension at hydrocarbon–water interfaces (e.g., heptane–water) [1]. Quantitative measurements showed that various globular proteins (e.g., bovine serum albumin, β-lactoglobulin, ovalbumin, and ribonuclease) reduce oil–water interfacial tension by approximately 8.5–17.1 mN/m, depending on protein type and concentration [2]. Modern reviews and systematic studies further confirm that proteins adsorb at oil–water interfaces, lower interfacial free energy, and promote emulsion formation and stability by forming viscoelastic interfacial films [3,4]. Protein adsorption has also

been shown to generate viscoelastic interfacial multilayers that significantly modify interfacial rheology and droplet stability [5]. Collectively, these studies provide robust experimental evidence that proteins in the aqueous phase reduce oil–water interfacial tension and alter interfacial mechanical properties, thereby facilitating droplet formation and stabilization in multiphase systems [6].

Interfacial rheology measurements (summary of previously published methodology [7]): Interfacial viscoelastic properties of aqueous phase–silicone oil systems were characterized in our previous study (ref [7]) using an oscillatory interfacial rheology approach. Briefly, measurements were performed at the interface between silicone oil (SO1000 and SO5000) and aqueous phases representing different compositions, including distilled water (DW), saline solution (SS), and model aqueous humor (MAH). Interfacial rheological measurements were carried out using an oscillatory shear protocol, in which the interface was subjected to controlled sinusoidal deformation. The complex interfacial modulus ($|G_i^*|$) and phase shift (δ) were determined as a function of shear deformation amplitude (γ) through amplitude sweep experiments. All measurements were conducted under controlled temperature conditions, and each experiment was repeated multiple times to ensure reproducibility. Prior to measurements, sufficient equilibration time was allowed to enable adsorption of surface-active species at the interface. The obtained interfacial rheological parameters provide quantitative information on the viscoelastic behavior of the interface, including its elastic and viscous contributions. Detailed experimental procedures, including instrument configuration and data analysis, are described in the original publication (ref [7]).

Supporting Tables

Table S1 Summary of key physicochemical findings from previous in vitro study on SO emulsification and phase behavior [7].

Parameter / Phenomenon	Experimental Method	Key Findings	Relevance to Present Study
**Phase behavior of SO–aqueous systems**	Controlled emulsification experiments with systematic oil/water ratios (1:9, 5:5, 9:1) under rotary agitation	Identified coexistence of W/O and O/W emulsions at balanced phase ratios, while asymmetric ratios produced single emulsion types (W/O at high oil fraction, O/W at high aqueous fraction). Droplet sizes ranged from micrometers to >100 μm depending on phase ratio	Supports conceptual framework that intraocular SO can exist as a dynamic multiphase system with locally varying phase continuity, enabling formation of both W/O and O/W microstructures in vivo
**Droplet size distribution and mass contribution**	Optical microscopy + ImageJ quantitative analysis	W/O droplets dominated mass fraction (>90% mass in large droplets), while O/W droplets were smaller and more numerous	Suggests W/O droplets can significantly affect optical and biological properties of SO despite lower number density
**Effect of aqueous phase composition**	Comparison of distilled water, salt solution, and protein-containing model aqueous humor	Inorganic salts had minor influence; proteins strongly altered interfacial properties and droplet size distribution	Supports biological relevance of proteins as dominant emulsifiers in intraocular environment
**Effect of temperature**	Emulsification at 20 °C and 40 °C	Temperature modified droplet size distributions only in protein-containing systems; higher temperature favored coarser W/O droplets due to strengthened interfacial films	Indicates physiological and inflammatory temperature variations can modulate droplet formation and stability
**Shear-driven emulsification**	Mechanical agitation (rotary shaking) under controlled hydrodynamic conditions	Emulsification required mechanical energy input; spontaneous emulsification was negligible. Shear promoted droplet formation and breakup	Supports shear-driven droplet generation by ocular movements and mechanical stresses in vivo
**Protein-mediated interfacial stabilization**	Model aqueous humor containing albumin and γ-globulins	Proteins strongly increased emulsion stability and promoted larger W/O droplets, while reducing O/W droplet formation	Demonstrates biological relevance of protein adsorption at intraocular SO–aqueous interfaces and supports protein-stabilized W/O droplets observed in vivo.
**Interfacial viscoelasticity**	BiCone interfacial rheometry (oscillatory shear)	Measured complex interfacial modulus (G*) and phase shift, showing protein adsorption transforms viscous interface into elastic viscoelastic films that resist droplet breakup	Provides mechanistic basis for droplet stability, deformation, and persistence during long-term tamponade

Table S2 Quantitative characterization of W/O droplets and intra-sample technical variability determined from six microscopy images analyzed for each porcine sample.

Sample	Condition	Images analyzed	Total droplets	Droplet diameter (µm) mean ± SD *	Droplets per image mean ± SD **	Image-level mean diameter (µm) mean ± SD ***
Pig 920 / 1300 mPa·s	Direct extraction	6	41	77.8 ± 98.3	6.8 ± 2.4	89.2 ± 54.2
	23-gauge	6	117	30.2 ± 26.7	19.5 ± 1.9	30.7 ± 11.2
	25- gauge	6	0	n.d.	n.d.	n.d.
Pig 920 / 5000 mPa·s	Direct extraction	6	77	63.6 ± 127.9	12.8 ± 6.6	84.3 ± 98.3
	23- gauge	6	10	71.6 ± 34.1	1.7 ± 1.2	56.8 ± 36.2
	25- gauge	6	0	n.d.	n.d.	n.d.
Pig 931 / 1300 mPa·s	Direct extraction	6	195	38.5 ± 45.6	32.5 ± 14.9	41.0 ± 25.9
	23- gauge	6	87	29.3 ± 39.3	14.5 ± 12.0	60.0 ± 52.9
	25- gauge	6	0	n.d.	n.d.	n.d.
Pig 931 / 5000 mPa·s	Direct extraction	6	90	46.0 ± 67.2	15.0 ± 7.6	50.6 ± 38.1
	23- gauge	6	11	44.0 ± 18.6	1.8 ± 1.0	42.9 ± 10.3
	25- gauge	6	30	17.7 ± 7.2	5.0 ± 2.4	19.9 ± 6.6

Notes: * Calculated from all individual droplets pooled from six microscopy images. ** Calculated from the number of droplets detected in each of the six microscopy images. *** Calculated from mean droplet diameters determined separately for each microscopy image. *All values represent technical (intra-sample) variability and spatial heterogeneity within individual samples rather than biological variation.*
Abbreviation: n.d. – not detected (no detected droplets)

Supporting Figures

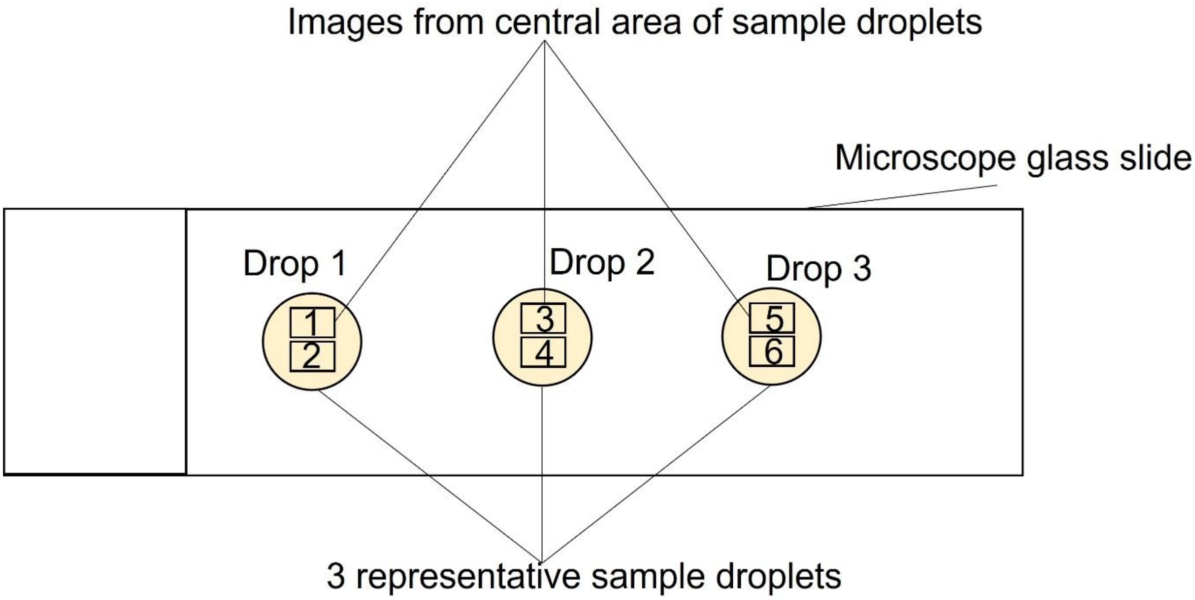

Figure S1 Schematic illustration of the sampling and imaging strategy used for droplet analysis. For each SO sample, three droplets of identical volume were placed on a microscope glass slide. From each droplet, two representative images were acquired from the central area, resulting in a total of six images per sample used for droplet size analysis.

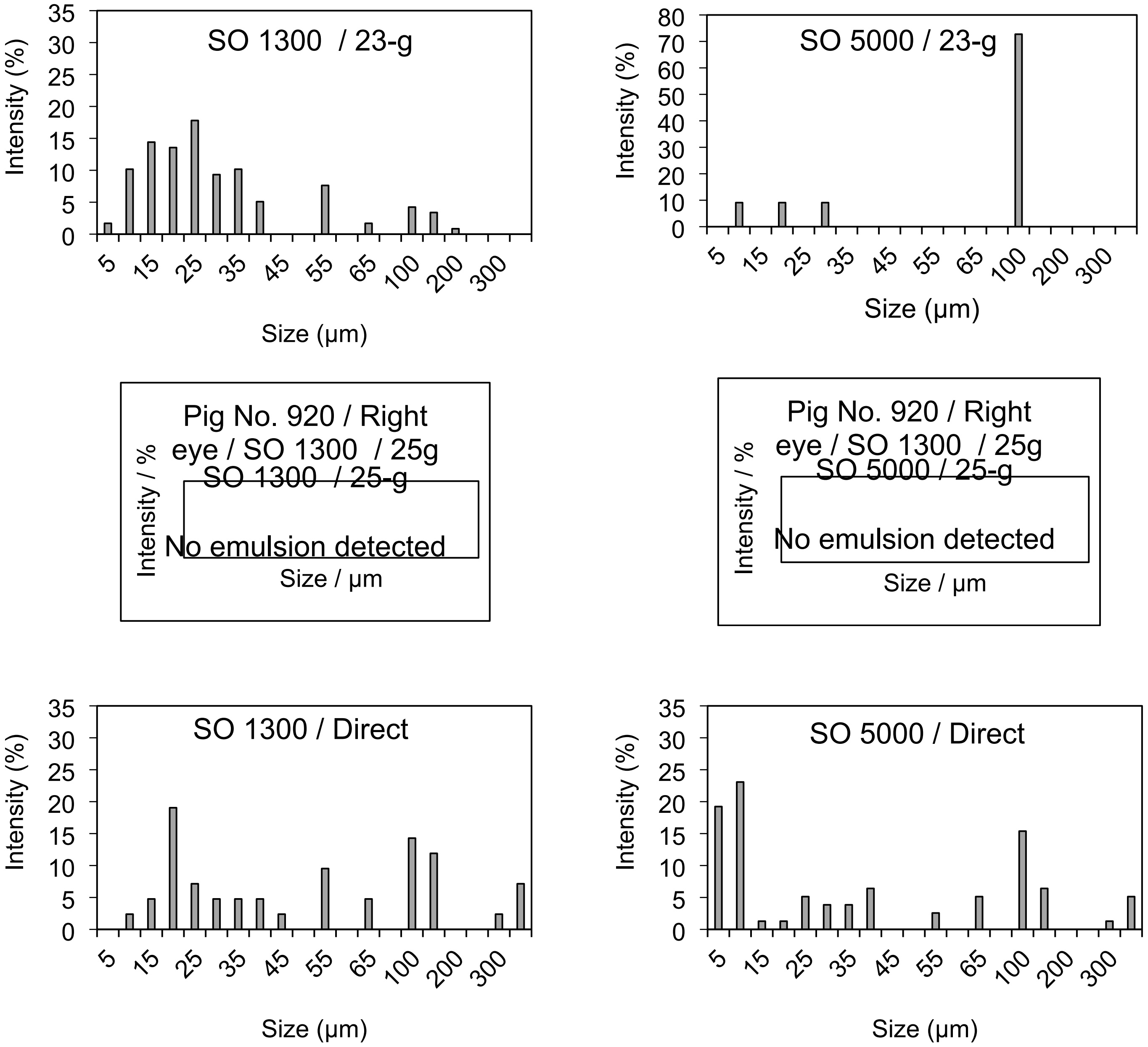

Figure S2 Complete droplet size distributions for animal No. 920 obtained by, 23-gauge aspiration, 25-gauge aspiration and direct extraction for both SO viscosities (1300 and 5000 mPa·s).

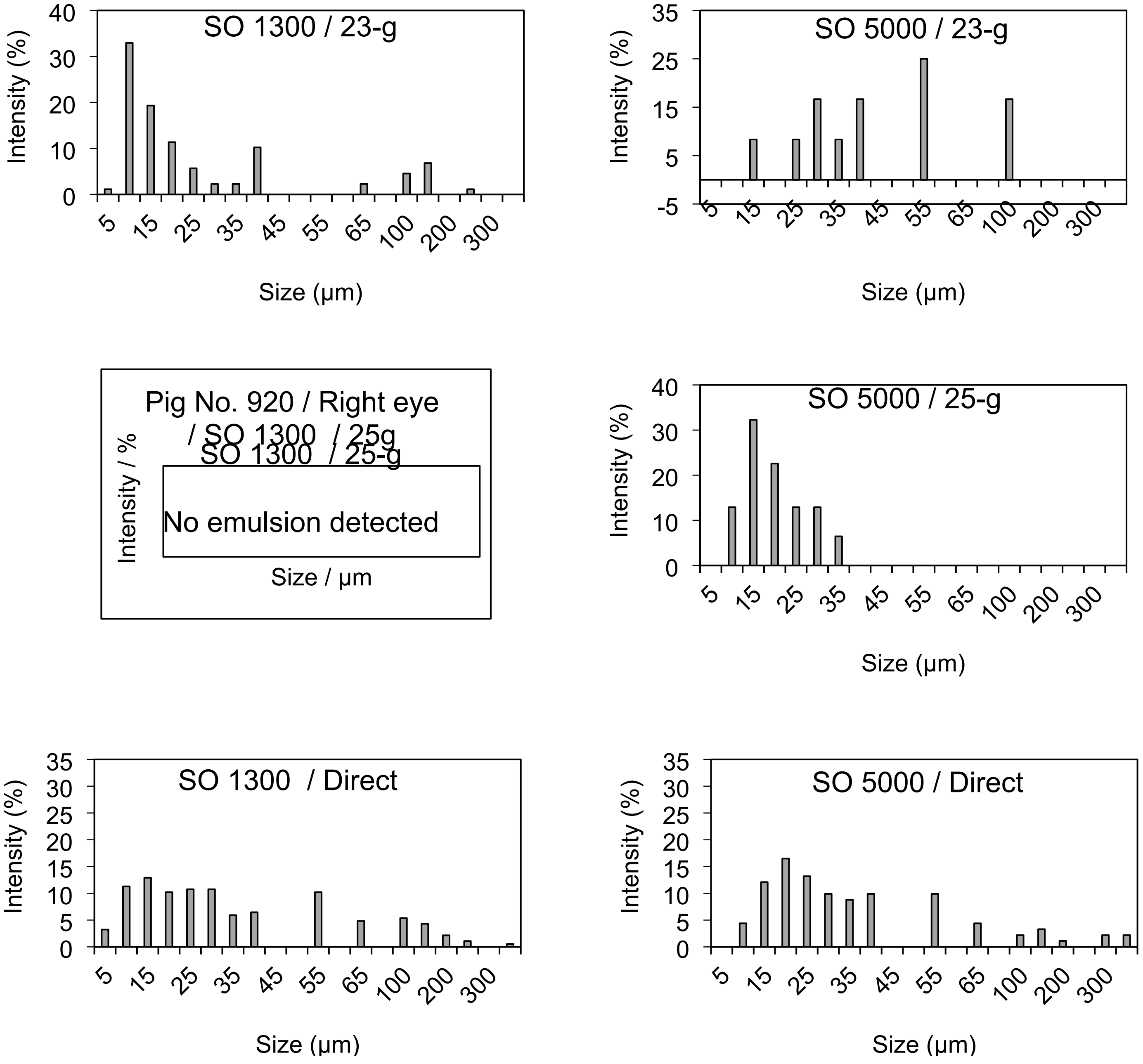

Figure S3 Complete droplet size distributions for animal No. 931 obtained by 23-gauge aspiration, 25-gauge aspiration and direct extraction for both SO viscosities (1300 and 5000 mPa·s).

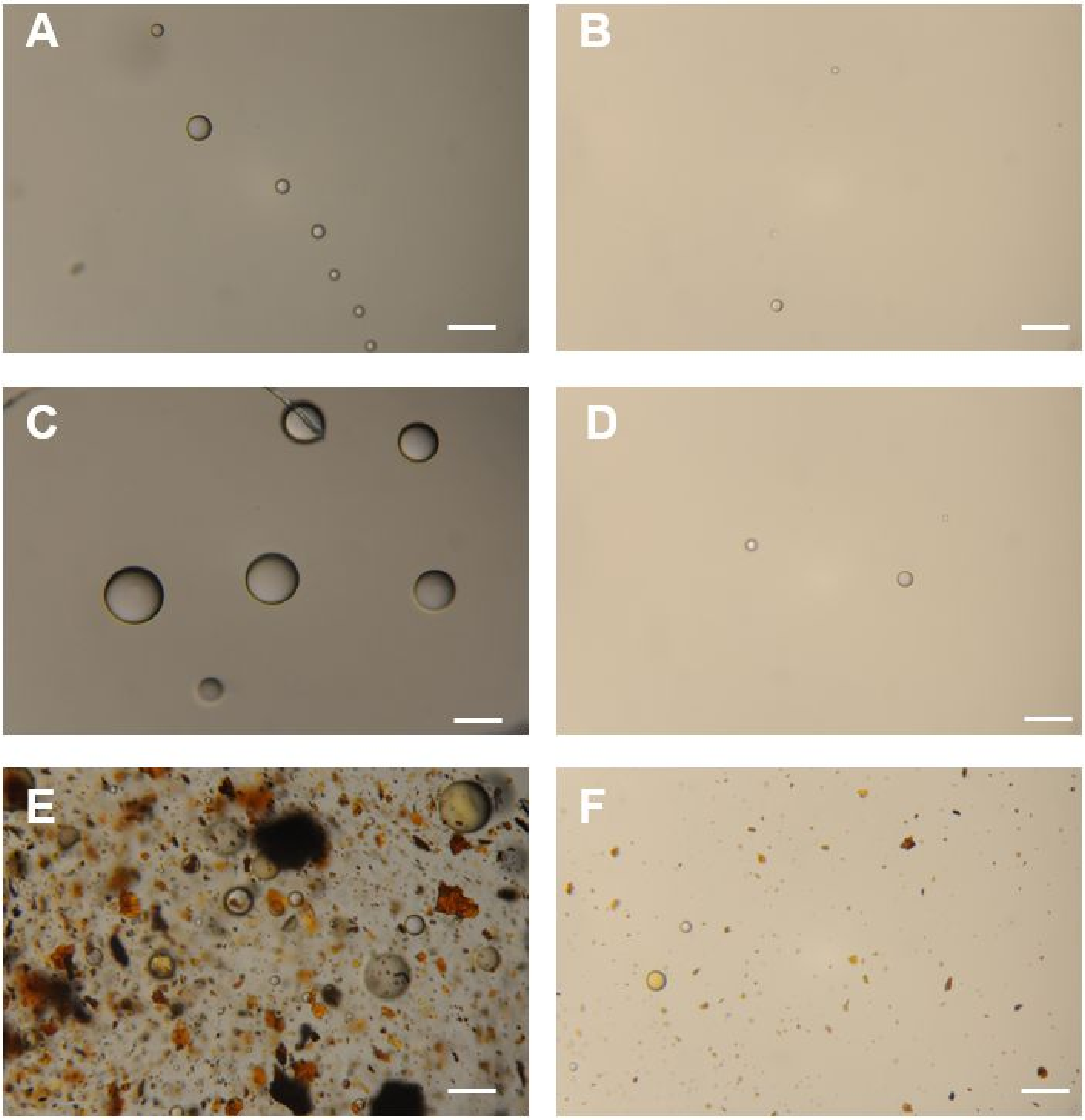

Figure S4 Representative microscopy images of W/O droplets detected in human SO explants. Patient 2, aspiration through a 25-gauge needle (A); Patient 6, aspiration through a 25-gauge needle (B); Patient 2, aspiration through a 23-gauge needle (C); Patient 6, aspiration through a 23-gauge needle (D). Methylene orange staining confirming the aqueous nature of droplets in the sample from Patient 2 (23-gauge aspiration) (E). Methylene orange staining confirming the aqueous nature of droplets in the sample from Patient 6 (23-gauge aspiration) (F). Scale bars = 100 µm.

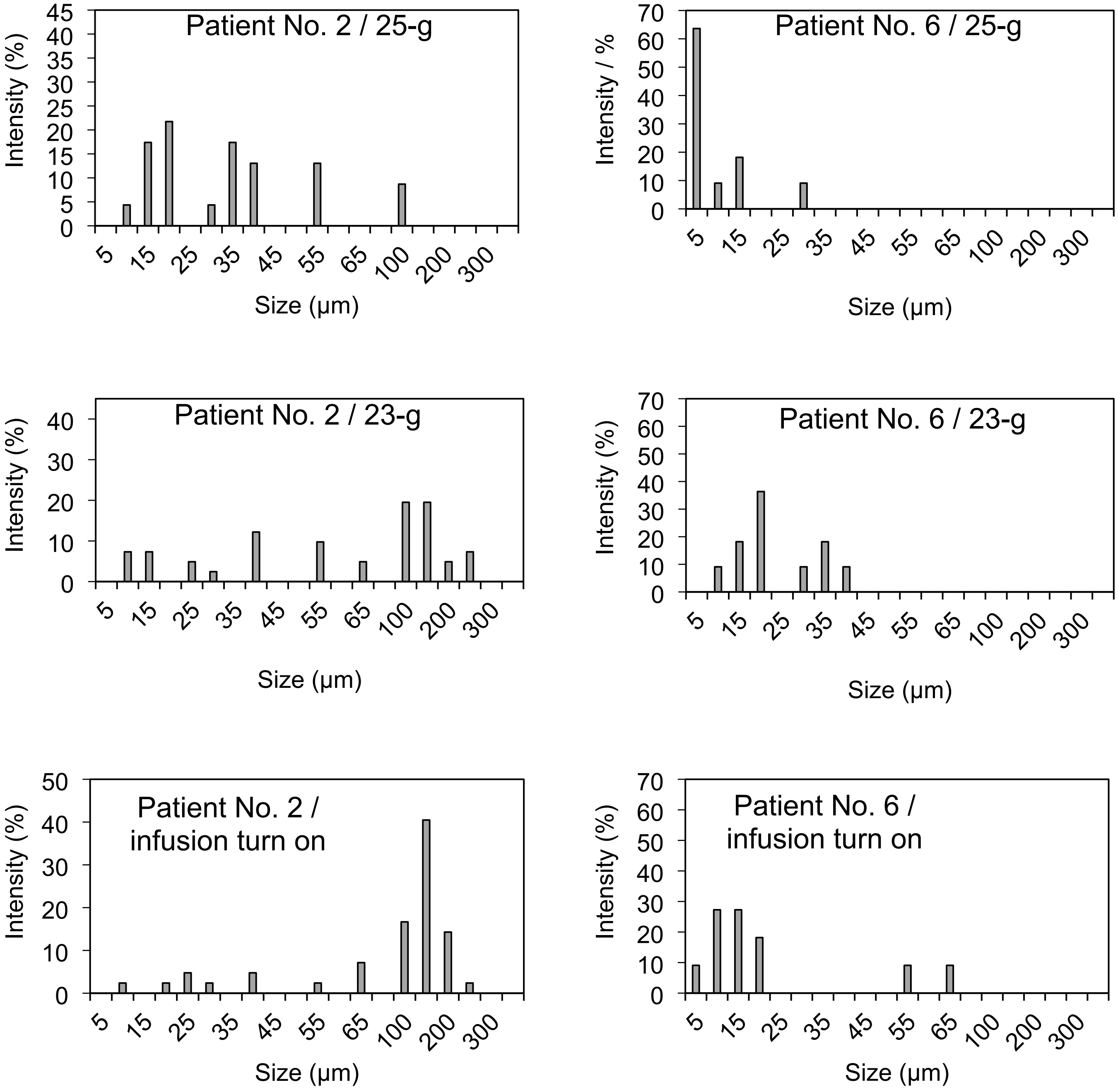

Figure S5 Complete droplet size distributions for patient No. 2 and No. 6 obtained by 23-gauge aspiration, 25-gauge aspiration and 25-gauge aspiration with infusion turn on.

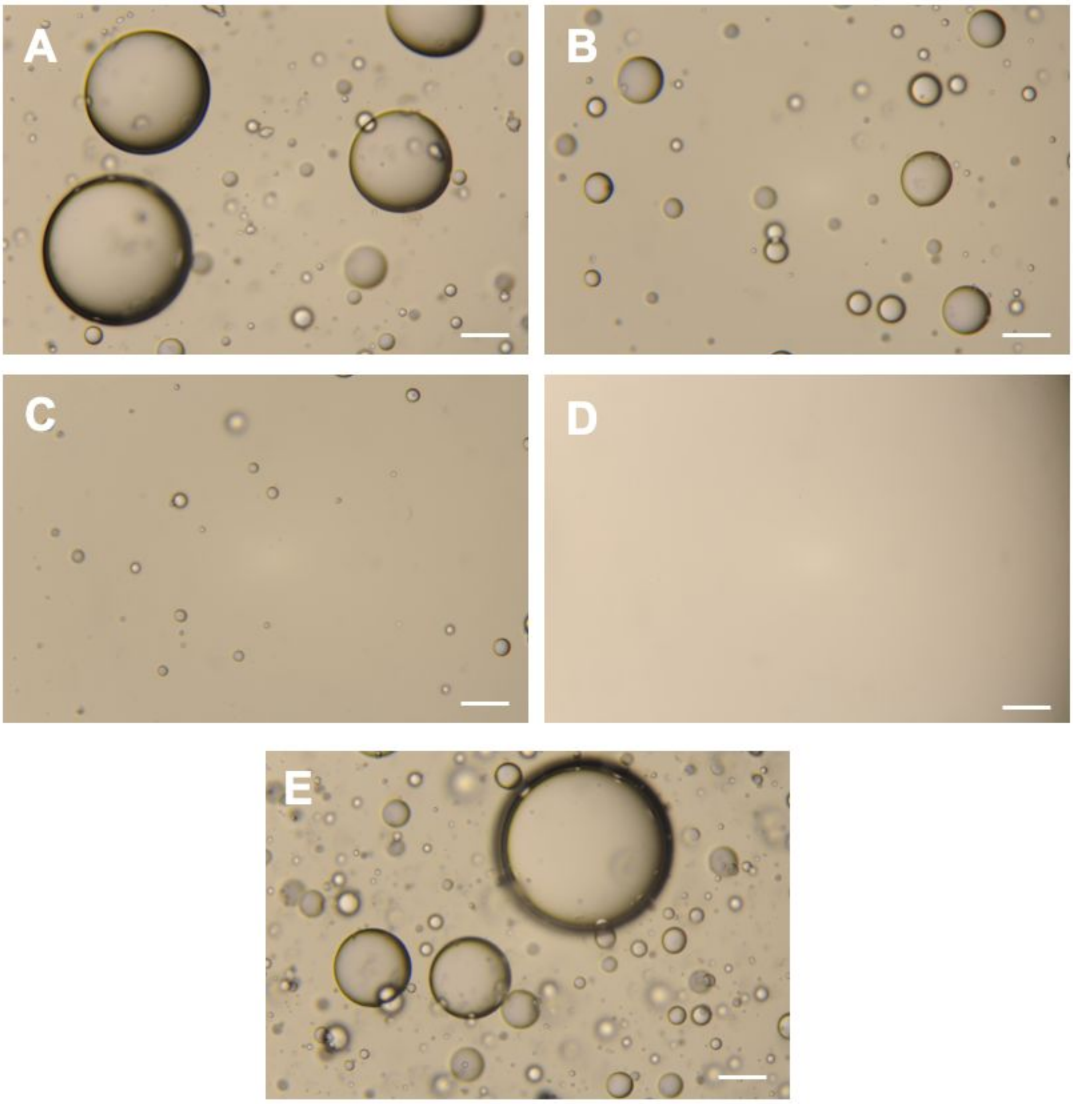

Figure S6 Representative microscopy images from control and in vitro experiments evaluating sampling-induced artefacts. Pre-formed W/O emulsion prior to aspiration (A). Emulsion after aspiration through a 23-gauge needle (B). Emulsion after aspiration through a 25-gauge needle (C). Pure SO aspirated through a needle without an aqueous phase (D). W/O droplets generated in the infusion-mimicking in vitro experiment (E). Scale bars = 100 μm.

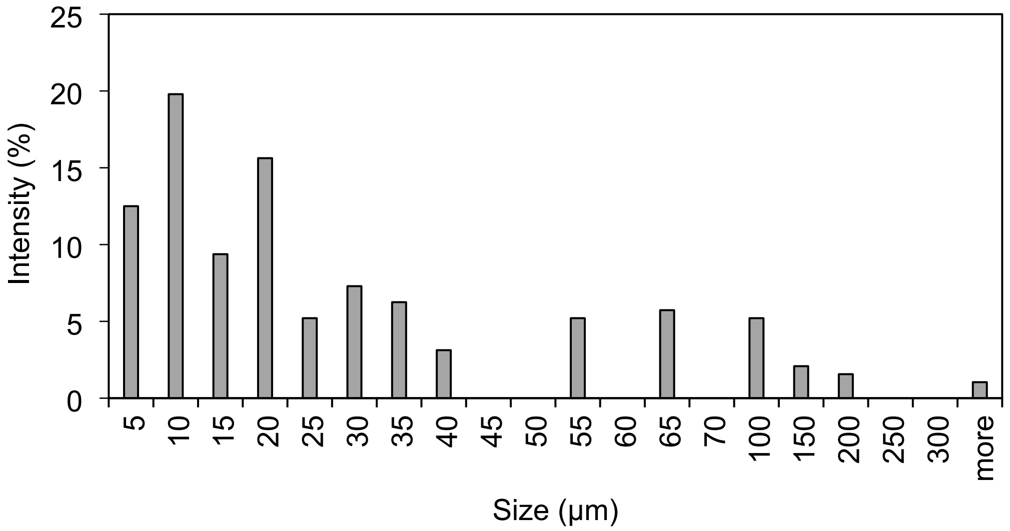

Figure S7 Droplet size distribution obtained in the infusion-mimicking in vitro experiment, showing a broad size range with the presence of large droplets.

Literature

(1) Beverung, C. J.; Radke, C. J.; Blanch, H. W. Protein Adsorption at the Oil/Water Interface: Characterization of Adsorption Kinetics by Dynamic Interfacial Tension Measurements. *Biophys. Chem.* **1999**, *81* (1), 59–80. https://doi.org/10.1016/S0301-4622(99)00082-4.

(2) Sah, H.; Choi, S.-K.; Choi, H.-G.; Yong, C.-S. Relation of Dynamic Changes in Interfacial Tension to Protein Destabilization upon Emulsification. *Arch. Pharm. Res.* **2002**, *25* (3), 381–386. https://doi.org/10.1007/BF02976643.

(3) Bergfreund, J.; Bertsch, P.; Fischer, P. Adsorption of Proteins to Fluid Interfaces: Role of the Hydrophobic Subphase. *J. Colloid Interface Sci.* **2021**, *584*, 411–417. https://doi.org/10.1016/j.jcis.2020.09.118.

(4) Lin, L.-H.; Bergfreund, J.; Fischer, P.; Bertsch, P. Plant Protein Adsorption at Oil–Water Interfaces: A Mapping Review Using Alternate Subphase Tensiometry. *Curr. Opin. Colloid Interface Sci.* **2025**, *77*, 101920. https://doi.org/10.1016/j.cocis.2025.101920.

(5) Baldursdottir, S. G.; Fullerton, M. S.; Nielsen, S. H.; Jorgensen, L. Adsorption of Proteins at the Oil/Water Interface—Observation of Protein Adsorption by Interfacial Shear Stress Measurements. *Colloids Surf. B Biointerfaces* **2010**, *79* (1), 41–46. https://doi.org/10.1016/j.colsurfb.2010.03.020.

(6) Nakamura, K.; Refojo, M. F.; Crabrree, D. V. *Factors Contributing to the Emulsification of Intraocular Silicone and Fluorosilicone Oils*; 1990; Vol. 31.

(7) Kamenická, B.; Pěnkavová, V.; Lyko Vachková, E.; Orvalho, S.; Zedníková, M.; Jaklová, N.; Stavárek, P.; Reháčková, M.; Rajs, P.; Veith, M.; Klusoň, P. Emulsification Complexity of Silicone Oil in Retinal Surgery: In Vitro Insights into Phase Behavior. *ACS Omega* **2026**. https://doi.org/10.1021/acsomega.5c09041.
